# Functional Delivery of Nanoimmunotherapies in Pancreatic Ductal Adenocarcinoma: A Barrier-Informed Pharmaceutics Framework

**DOI:** 10.3390/pharmaceutics18101305

**Published:** 2026-10-15

**Authors:** Baodan Xu, Dan Wei, Kaishan Tao, Yang Wang

**Affiliations:** 1 School of Medicine, Northwest University, Xi’an 710069, China; 2 Department of Hepatobiliary Surgery, Air Force Medical University, Xi’an 710032, China

**Keywords:** pancreatic ductal adenocarcinoma, nanoimmunotherapy, nanomedicine, drug delivery, formulation design, functional delivery, pharmacokinetics, critical quality attributes, translational pharmaceutics, biomarkers

## Abstract

Pancreatic ductal adenocarcinoma (PDAC) presents a stringent drug-delivery environment in which tumor accumulation does not necessarily translate into target-cell exposure or cargo activity. This critical narrative review develops a barrier-informed framework for evaluating nanoimmunotherapies across systemic exposure, tumor access, target-cell delivery, cargo release, target engagement, and pharmacological activity. The framework links vascular, stromal, immune, metabolic, and intracellular barriers to cargo–carrier selection, administration route, biological identity, pharmacokinetics, biodistribution, and intracellular trafficking. Platforms discussed include nanovaccines and nucleic-acid therapeutics, engineered extracellular vesicles, nanoengineered oncolytic-virus delivery, nanoparticle-assisted immune-cell programming, checkpoint modulation, and stromal or metabolic strategies relevant to immune-directed therapy. Functional-delivery evidence is integrated with critical quality attributes, mechanism-based potency, manufacturing comparability, biosafety, repeat-dose performance, and biomarker-guided clinical development. We propose a qualitative stage-gating framework based on concordant evidence of target-site exposure, target-cell activity, reproducible product quality, acceptable repeat-dose performance, and a testable biomarker strategy. This framework supports formulation selection, cross-platform comparison, and translational prioritization of PDAC nanoimmunotherapies.

## 1. Introduction

Pancreatic ductal adenocarcinoma (PDAC) remains one of the most lethal solid malignancies, with incidence closely paralleling mortality [1,2]. Although FOLFIRINOX and gemcitabine plus nanoparticle albumin-bound paclitaxel are established treatments for advanced disease [3,4], durable responses remain limited. Dense desmoplasia, vascular compression, heterogeneous perfusion, immune exclusion, and metabolic stress collectively restrict therapeutic delivery and antitumor immunity [5,6], making PDAC a challenging setting for immune-directed drug delivery. Nanocarriers can improve cargo stability, pharmacokinetics, biodistribution, cellular uptake, and controlled release [7]. However, increased tumor accumulation or prolonged circulation does not necessarily translate into pharmacological activity. Protein corona formation, phagocytic sequestration, stromal restriction, and intracellular trafficking can alter nanocarrier fate and limit productive cargo delivery [7,8].

In this review, nanoimmunotherapy refers to immune-directed therapeutic strategies in which an engineered nanoscale component directly contributes to cargo delivery, localization, release, or immune activation. This definition includes nanocarrier-mediated immunomodulation, nanovaccines, engineered extracellular vesicles, nanoengineered oncolytic-virus delivery, and nanoparticle-assisted immune-cell programming. Related approaches without a direct nanoscale contribution to immune function are discussed as supporting evidence rather than core nanoimmunotherapy platforms.

Functional delivery is defined here as progression from target-site exposure and cellular access to cargo availability, target engagement, and biological activity. It should be assessed using complementary measures of distribution, cell-specific delivery, intracellular activity, and mechanism-linked pharmacological responses. Recent reviews have summarized nanocarrier platforms, immune targets, and tumor-microenvironment-responsive strategies in PDAC [9,10,11,12]. However, biological barriers, formulation design, functional delivery assessment, pharmaceutical quality, and translational development are often considered separately. Here, we integrate these domains in a barrier-informed pharmaceutics framework linking PDAC-associated barriers to cargo–carrier selection, administration route, biological identity, pharmacokinetics, biodistribution, intracellular trafficking, and therapeutic activity. This framework supports evaluation of nanoimmunotherapy platforms and translational development.

## 2. Literature Search Strategy and Evidence Selection

A structured literature search was conducted in PubMed and Web of Science for publications available up to 31 August 2026. Multiple complementary searches combined terms related to PDAC, biological barriers, nanomedicine and drug delivery, immunotherapy, extracellular vesicles, nucleic-acid therapeutics, oncolytic viruses, immune-cell engineering, and translational pharmaceutics. Search concepts, representative Boolean combinations, database-adapted strategies, evidence-selection procedures, and limitations of the available search documentation are detailed in Supplementary Table S1.

The retrieved records were consolidated into a bibliographic dataset of 7341 records, of which 6383 remained after cross-search deduplication. Title-level screening retained 5148 records, and subsequent assessment identified 1175 as potentially relevant to the barrier-informed pharmaceutics framework. As this was a critical narrative rather than a systematic review, publications were subsequently selected according to their relevance to the mechanistic, pharmaceutical, and translational issues addressed. Records were included when they addressed PDAC-associated delivery barriers, nano-enabled or immune-directed therapeutic strategies, functional delivery, or directly relevant pharmaceutical and translational considerations; records without relevance to these predefined domains were excluded.

Additional publications were included when needed to support clinical background, methodological considerations, or broadly applicable pharmaceutical and translational principles. Of the 121 publications cited, 69 originated from the database-derived literature pool and 52 from supplementary evidence selection.

## 3. PDAC as a Multilevel Barrier to Drug Delivery

### 3.1. Vascular and Perfusion Barriers

PDAC is characterized by dense desmoplasia, vascular compression, elevated interstitial pressure, and heterogeneous perfusion, which restrict extravasation and intratumoral distribution [6,13,14,15,16]. CAF heterogeneity further generates spatial variation in matrix architecture and tissue transport [13,14,17,18]. These barriers affect therapeutic modalities differently: antibodies diffuse slowly, oncolytic viruses may remain near accessible regions, immune-cell trafficking is restricted, and nanocarriers often localize near perfused vessels or tumor margins rather than penetrating malignant-cell-rich compartments [6,13,14]. Thus, systemic circulation alone does not ensure adequate tumor exposure, which should be evaluated in relation to lesion distribution, vascular proximity, stromal penetration, and target-cell access.

### 3.2. Stromal and Cellular Barriers

The PDAC stroma is both a physical barrier and an active regulator of immunity. CAF-derived CXCL12, IL-6, TGF-β, and matrix-associated signaling restrict T-cell access and promote immunosuppression [13,17,18,19,20,21,22]. Because CAF populations are functionally heterogeneous, selective stromal normalization may be preferable to indiscriminate stromal depletion [23,24]. Myeloid cells further influence nanomedicine disposition: tumor-associated macrophages can sequester nanoparticles in the liver, spleen, and tumor, but may also serve as therapeutic targets or local cargo reservoirs when uptake produces functional reprogramming [7,8]. Myeloid-derived suppressor cells and regulatory T cells additionally constrain immune activation [19,25]. Thus, stromal and myeloid populations can simultaneously act as delivery barriers, therapeutic targets, and determinants of nanocarrier disposition.

### 3.3. Metabolic and Intracellular Barriers

Poor perfusion and high metabolic demand generate hypoxia, acidosis, and nutrient competition in PDAC [6,13]. HIF-1α-dependent adaptation promotes glycolysis, lactate production, PD-L1 expression, and impaired antigen presentation [26,27,28,29,30], while acidosis and hypoxia-driven CD39–CD73 signaling suppress cytotoxic T- and natural killer-cell function [31,32]. Altered glucose, glutamine, tryptophan, and lipid metabolism further contributes to tumor progression and immune dysfunction [28,30,33,34,35,36,37,38,39]. These metabolic features may also provide formulation triggers. Low pH, reactive oxygen species, and elevated enzyme activity can enable stimulus-responsive cargo release, although heterogeneous conditions across primary and metastatic PDAC may limit trigger reliability [7,8]. Because metabolic pathways are shared among malignant, stromal, and immune cells, such interventions require sufficient cell-type selectivity to preserve antitumor immunity. For intracellularly active cargos, endosomal or lysosomal sequestration, premature release, and cargo degradation can limit productive intracellular availability [7,8,40].

### 3.4. Translating Biological Barriers into Formulation Requirements

**Figure 1:**
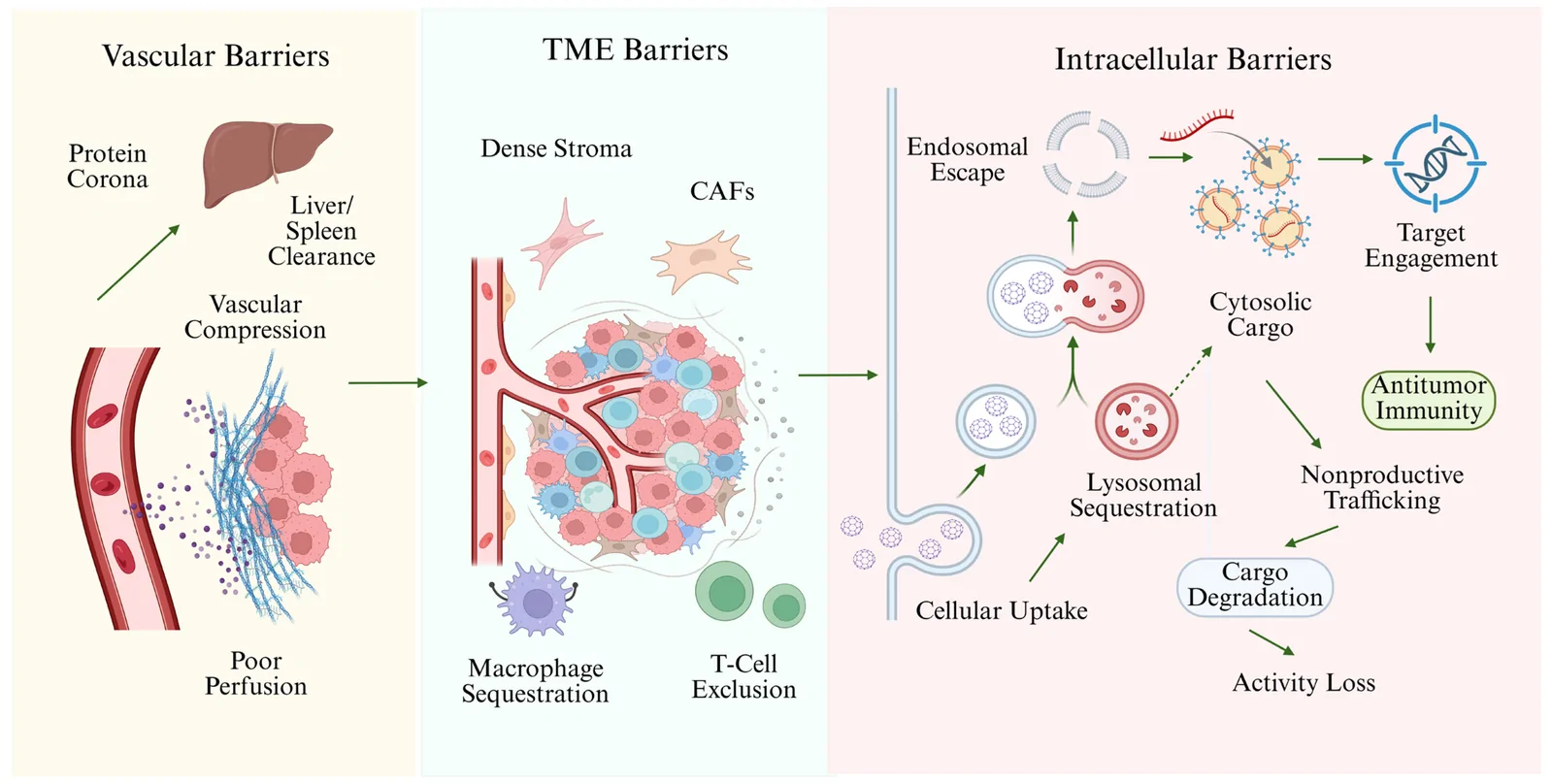
Sequential barriers to functional nanoimmunotherapy delivery in pancreatic ductal adenocarcinoma. Arrows indicate the directional progression of delivery and intracellular trafficking, including productive and nonproductive pathways.

PDAC-associated barriers create a sequential delivery problem: systemic exposure must lead to vascular access, extravasation, stromal penetration, target-cell delivery, access to the required pharmacological compartment, cargo release, target engagement, and biological activity [7,8,40]. Formulation design and evaluation should therefore be matched to the dominant barrier and intended site of action, with consideration of lesion- and cell-specific exposure, mechanism-specific pharmacological endpoints, and repeat-dose effects on clearance and biodistribution [41]. Standardized characterization and reporting are also needed to distinguish formulation effects from differences in models, dosing, and analytical methods [42]. Figure 1 summarizes the major barriers across this delivery sequence, and Table 1 links them to formulation implications, recommended readouts, and potentially misleading surrogate endpoints.

## 4. Barrier-Informed Formulation Design for Functional Delivery

### 4.1. Cargo–Carrier Matching

Nanocarrier selection should reflect cargo stability, site of action, release requirements, and administration route [7]. mRNA and siRNA require protection from degradation, efficient encapsulation, cellular uptake, endosomal escape, and cytosolic release, making ionizable lipid nanoparticles particularly suited to these requirements [44,46]. Proteins, antibodies, and enzymes require preservation of structural integrity and biological activity during formulation, storage, administration, and release, whereas viral carriers must retain infectivity, replication competence, and tropism [47,48]. For poorly soluble small molecules and metabolic inhibitors, formulation should control loading, free-drug fraction, release kinetics, and systemic exposure [45,49]. Inorganic materials are most justified when their intrinsic physicochemical properties contribute directly to therapeutic function [50,51,52,53], whereas biomimetic and hybrid systems require additional control of composition, biological identity, and batch consistency [54,55,56].

### 4.2. Administration Route and Disease Distribution

Administration route should be matched to disease distribution and the intended pharmacological compartment. Intravenous administration is most relevant to disseminated PDAC but exposes formulations to plasma proteins, complement, antibodies, phagocytic clearance, and heterogeneous tumor perfusion. Systemic development therefore requires quantitative pharmacokinetics and evidence of delivery to both primary and metastatic lesions [7,8]. Intratumoral administration provides high local exposure but may produce heterogeneous distribution within fibrotic tumors and has limited relevance to distant metastases. Intraperitoneal delivery may be appropriate for regional or peritoneal disease, whereas postoperative depots primarily target residual disease at the resection site. For vaccines, delivery to lymphoid tissues and antigen-presenting cells may be more important than direct pancreatic accumulation [44,57,58]. Route selection should therefore be regarded as an integral formulation variable rather than a procedural consideration.

### 4.3. Biological Identity and Protein Corona

The biological behavior of a nanocarrier cannot be predicted solely from its manufactured properties. Following administration, adsorption of proteins and other biomolecules generates an acquired biological identity that can alter particle size, surface properties, colloidal stability, ligand accessibility, immune recognition, biodistribution, cellular uptake, and intracellular trafficking [43,59]. Protein-corona formation can redirect nanocarriers toward lysosomal sequestration or uptake by non-target phagocytes [40]. In PDAC, tumor-associated particle signals may therefore reflect macrophage sequestration rather than delivery to malignant, stromal, dendritic, or effector immune cells. Surface modification also influences biological identity during repeated administration. PEGylation can improve colloidal stability and circulation, but its effects depend on polymer density, molecular weight, anchoring chemistry, shedding, administration route, and dosing schedule. Pre-existing or treatment-induced anti-PEG antibodies may promote complement activation, accelerated clearance, particle disruption, and reduced cargo delivery [41]. Alternative coatings may reduce these effects but do not eliminate protein adsorption or immune recognition.

### 4.4. Active Targeting and Target Accessibility

Active targeting operates within the constraints of tumor exposure and tissue accessibility. Targeting ligands may enhance receptor binding, internalization, or retention after a nanocarrier reaches an accessible target but do not directly overcome poor perfusion, vascular compression, limited extravasation, or stromal resistance [7,8]. Targeting performance is further influenced by target accessibility, internalization kinetics, recycling, shedding, intracellular trafficking, and, for antibody-targeted systems, ligand density, orientation, surface accessibility, retained affinity, and corona-mediated masking [60,61]. Compositionally matched non-targeted controls should therefore be used to determine the effects of targeting on cellular distribution, intracellular trafficking, and pharmacological activity.

### 4.5. Intracellular Trafficking, Cargo Release, and Functional Delivery

**Figure 2:**
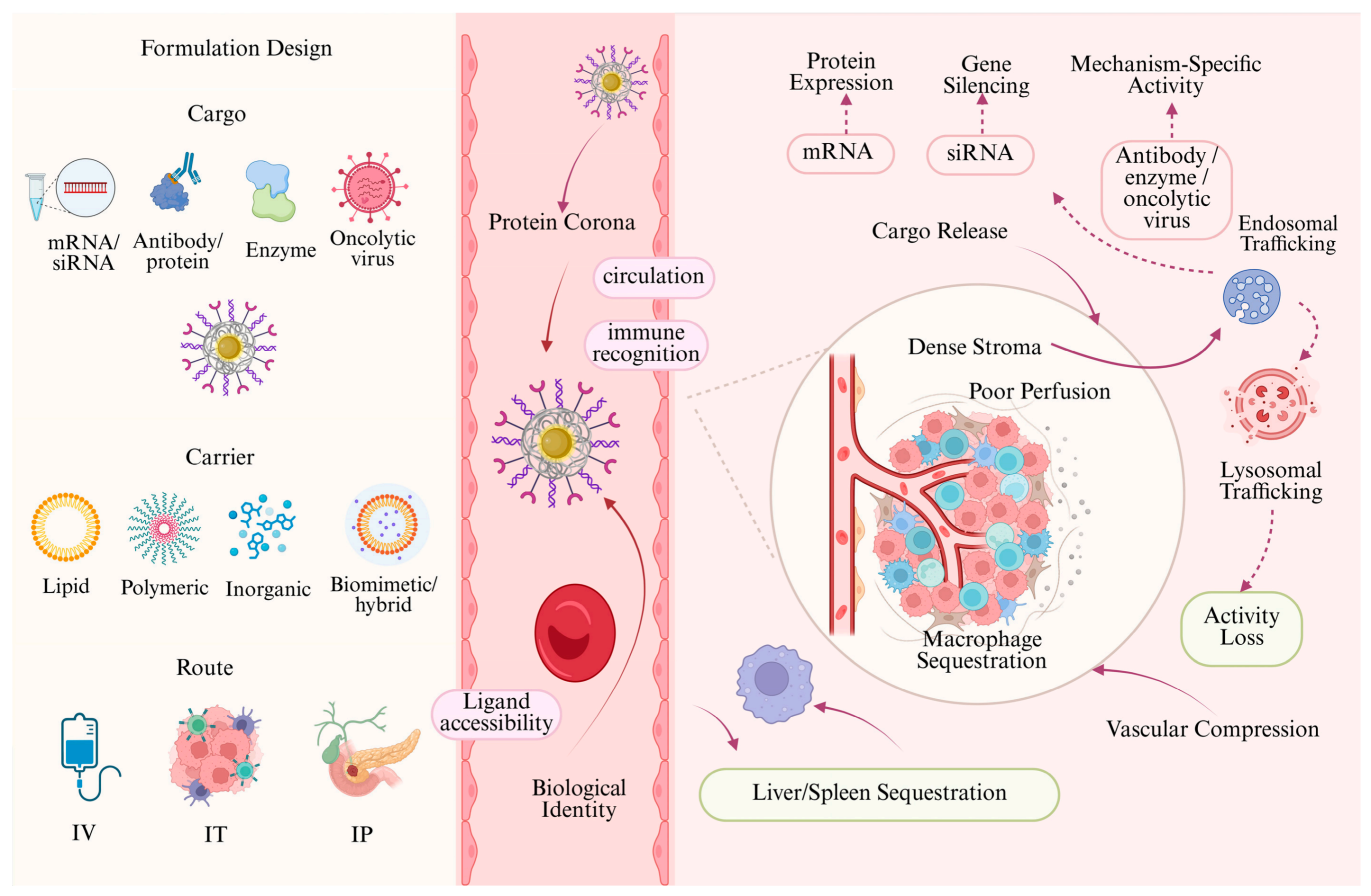
The formulation-to-function continuum in PDAC nanoimmunotherapy: cargo–carrier–route matching, acquired biological identity, tumor barriers, and mechanism-linked activity. Arrows indicate the directional progression of nanocarrier disposition, cargo delivery, intracellular trafficking, and functional outcomes, with branching pathways representing alternative productive or nonproductive fates.

After reaching the intended cell population, a formulation must deliver active cargo to the appropriate pharmacological compartment and enable target engagement. These requirements are cargo-specific: mRNA and siRNA require cytosolic availability followed by protein expression or gene silencing [44,46]; antibodies require preserved binding activity; enzymes require substrate access and catalytic activity; and oncolytic viruses must retain infectivity and productive replication [47,48]. Formulation optimization should therefore use cargo-specific pharmacodynamic endpoints linking cargo disposition to therapeutic function [40]. Figure 2 illustrates the formulation-to-function continuum from cargo–carrier–route selection and acquired biological identity through tumor barriers and intracellular trafficking to pharmacological activity.

## 5. Nanoimmunotherapy Platforms: From Formulation to Function

### 5.1. Nanoengineered Oncolytic Virus Delivery

Nanoengineered oncolytic-virus delivery systems are considered nanoimmunotherapy when an engineered nanoscale component directly contributes to viral protection, localization, controlled delivery, or immune-directed function. Oncolytic viruses themselves combine selective tumor-cell lysis with immune stimulation through antigen release, inflammatory signaling, and immune-cell recruitment [47,62]. In PDAC, however, systemic administration is constrained by neutralizing antibodies, hepatic sequestration, phagocytic clearance, inefficient tumor penetration, and dense stromal architecture. Nanoengineering approaches can address these barriers by modifying viral pharmacokinetics and surface interactions, protecting viral particles, altering biodistribution, or enabling controlled delivery while preserving infectivity and replication competence [48,63,64,65,66]. Representative strategies include polymer-coated adenoviruses, nanoparticle-associated viral delivery systems, and vesicular or cell-mediated carriers in which nanoscale engineering contributes directly to viral transport or protection [48,63,64,65,66]. Clinically evaluated oncolytic viruses such as VCN-01 and LOAd703 remain useful translational benchmarks for stromal modulation and immune activation, but they should not be classified as core nanoimmunotherapy platforms unless a nanoscale component directly contributes to therapeutic delivery or function [62,67]. Accordingly, functional evaluation of nanoengineered viral systems should extend beyond viral genome detection or tumor localization. Relevant endpoints include preserved infectivity, productive replication, transgene expression, and intercellular viral spread after delivery. Route-dependent biodistribution, post-release viral activity, immunogenicity, and reproducible virus-material composition should also be characterized because these parameters determine whether nanoscale engineering improves productive viral delivery rather than particle accumulation alone [47,48,63].

### 5.2. Nanovaccines and Nanocarrier-Mediated Nucleic-Acid Immunomodulation

Nanovaccines and nanocarrier-mediated nucleic-acid therapeutics qualify as nanoimmunotherapy when the nanoscale formulation directly enables immune priming, immune modulation, or delivery of an immune-active cargo to the relevant cellular compartment. These systems require delivery to defined immune or tumor-associated cell populations and appropriate intracellular sites of action [44,46,68,69,70]. In PDAC, impaired antigen presentation, limited T-cell infiltration, myeloid immunosuppression, antigen heterogeneity, and stromal exclusion restrict immune activation. RNA cargos require protection from degradation, efficient cellular uptake, endosomal escape, and sufficient cytosolic availability [44,46]. Lymphoid-targeted formulations can enhance immune priming, whereas tumor- or pancreas-directed systems can localize nucleic-acid expression in malignant, stromal, or resident immune cells. Pancreas-tropic ionizable lipid nanoparticles have enabled local mRNA expression after intraperitoneal administration [71,72], while spleen-targeted KRAS^G12D^ mRNA vaccination combined with irinotecan-loaded silicasomes integrated immune priming with tumor-localized immunogenic cell death [73]. Other nano-enabled systems have delivered STING or TLR agonists and pyroptosis-inducing mRNA cargos to promote antitumor immunity [74,75,76]. Personalized RNA vaccination has generated neoantigen-specific T-cell responses in a subset of patients with resected PDAC [77,78]. Functional evaluation should link formulation delivery to antigen expression or gene regulation, antigen presentation, immune-cell activation, tumor infiltration, and effector function. Antigen heterogeneity, transient expression, off-target delivery, immune toxicity, and manufacturing complexity remain translational challenges [44,46,77,78].

### 5.3. Nanoparticle-Assisted In Vivo Immune-Cell Programming

Conventional CAR-based cell therapies provide important translational context but are not inherently nanoimmunotherapies. CAR-based approaches targeting mesothelin, CLDN18.2, FAP, CXCR4, and c-MET have demonstrated activity against malignant or stromal compartments in PDAC, while remaining limited by antigen heterogeneity, stromal exclusion, immune suppression, metabolic constraints, and cellular exhaustion [79,80,81,82,83]. Nanoimmunotherapy approaches instead use nanoscale systems to modify engineered immune cells or directly program endogenous immune populations [84]. Examples include collagenase-containing nanogels associated with CAR-T cells to improve stromal penetration [85]. CD5-targeted lipid nanoparticles delivering FAP-CAR mRNA to generate transient CAR-T cells in vivo [86], and intraperitoneal mRNA lipid nanoparticles enabling macrophage CAR programming and antitumor activity [87]. Together with related targeted CAR-mRNA delivery studies, these platforms demonstrate the feasibility of nanoparticle-mediated immune-cell engineering while emphasizing the need to control cell selectivity, expression kinetics, and functional persistence [84,88]. The pharmaceutical requirements differ from those of conventional ex vivo cell products. Ex vivo CAR therapies depend substantially on cell-manufacturing quality attributes [89], whereas in vivo programming requires controlled nanoparticle biodistribution, selective nucleic-acid delivery, intracellular RNA release, receptor-expression kinetics, and functional activity of programmed host cells [84,88]. Off-target transfection, expression duration, repeat-dose performance, systemic inflammation, and product consistency therefore require dedicated evaluation.

### 5.4. Nano-Enabled Checkpoint Modulation and Immune Combination Therapy

Immune checkpoint blockade is not inherently nanotechnology-based; here, nano-enabled combinations are included when the nanoscale component directly contributes to localization, release, co-delivery, or immune pharmacology. In PDAC, low immunogenicity, defective antigen presentation, restricted T-cell infiltration, stromal resistance, and myeloid suppression limit responses to checkpoint blockade. Representative systems include nanogels co-delivering anti-PD-L1 and pirfenidone [90], bacteria-associated nanoscale platforms combining collagenase with anti-PD-L1 [91], ultrasound-responsive nanoparticles delivering PD-L1 inhibitors and nitric oxide [92], and photothermal systems combining PD-L1 and CXCR4 inhibition [93]. Nucleic-acid formulations have coupled neoantigen vaccination with local expression of checkpoint-binding proteins [94], while enzyme-responsive co-delivery of PD-1 and TGF-β pathway inhibitors illustrates spatially coordinated immunomodulation [95]. For combination products, coordinated functional delivery requires independent evidence of each cargo’s stability, release, exposure, and pharmacological activity. Dose ratio, spatial colocalization, trigger accessibility, and systemic exposure should be evaluated together with intratumoral pharmacodynamic responses to determine whether the intended combination mechanism is preserved in vivo.

### 5.5. Extracellular Vesicles as Biologically Derived Nanoimmunotherapy Platforms

Extracellular vesicles (EVs) are biologically derived nanocarriers capable of transporting nucleic acids, proteins, and other bioactive molecules. Their cargo-protective properties and engineering potential support therapeutic delivery, but source-cell variability, isolation procedures, and engineering strategies introduce product heterogeneity [96]. In PDAC, EV-mediated KRAS^G12D^ RNA interference provides an example of functional nucleic-acid delivery. Mesenchymal-cell-derived iExosomes carrying KRAS^G12D^-targeting siRNA or shRNA showed preclinical activity and highlighted challenges in source-cell control, cargo loading, purification, and standardization [97]. In a phase I study, KRAS^G12D^-targeting siRNA-loaded exosomes were well tolerated and showed exploratory evidence of KRAS-pathway modulation and increased intratumoral CD8^+^ T-cell infiltration; complementary preclinical findings suggested enhanced antitumor activity when combined with CTLA-4 blockade [98]. Because KRAS silencing is not inherently immunotherapeutic, its relevance here lies in immune-directed combination strategies. Li et al. developed photoresponsive exosomes incorporating a STING agonist, enabling light-triggered cytoplasmic delivery, STING activation, and enhanced antitumor immune responses in preclinical PDAC models [99]. Clinical evidence for EV-mediated immunotherapy in PDAC remains preliminary. Translation of EV-based nanoimmunotherapies requires control of source-cell characteristics, purity, cargo-loading efficiency, dose normalization, manufacturing scalability, storage stability, and batch-to-batch reproducibility. The MISEV2023 guidelines emphasize standardized characterization and appropriate controls to distinguish EV-associated components from non-vesicular contaminants [56]. Therapeutic development requires assessment of cargo integrity, loading efficiency, biodistribution, target-cell delivery, immune recognition, and repeat-dose safety. Mechanism-linked potency assays should quantify functional outcomes such as KRAS silencing, STING activation, and downstream immune responses.

### 5.6. Supporting Nanotherapeutic Strategies Relevant to Immune Modulation

**Figure 3:**
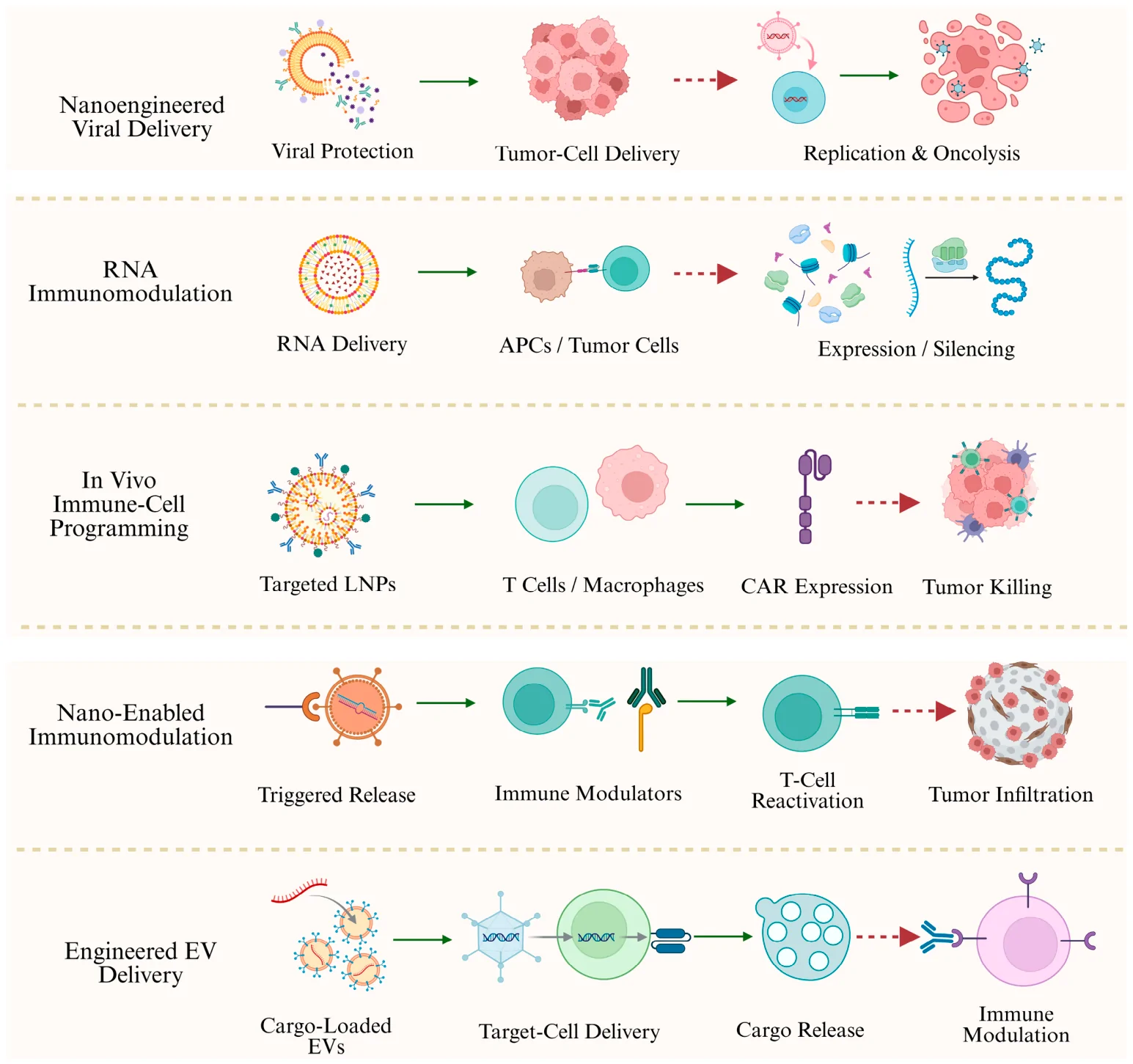
Nano-enabled therapeutic modalities and their mechanism-linked functional outcomes in PDAC. Solid green arrows indicate sequential delivery or mechanistic steps, whereas dashed red arrows indicate downstream functional outcomes.

Stromal and metabolic nanotherapies are considered supporting strategies rather than core nanoimmunotherapy when their primary mechanism is physical or metabolic modulation without a demonstrated immune-directed contribution. They remain relevant to a barrier-informed framework because stromal architecture and metabolic stress influence tissue penetration, immune access, and responsiveness to immunotherapy. Stromal modulation should improve perfusion and tissue access while preserving tumor-restraining stromal functions; given CAF heterogeneity, selective normalization is preferable to indiscriminate depletion [17,18,23,24]. Stromal remodeling can also enable immune-directed platforms, including CAR-T-associated nanogels, antifibrotic checkpoint formulations, and collagenase-containing bacteria-associated systems [85,90,91]. Improved perfusion or matrix depletion does not itself establish therapeutic benefit, as illustrated by the failure of hyaluronan depletion to improve survival in the HALO 301 trial [100]. Metabolic nanotherapies may influence the immune context of PDAC by altering nutrient competition, hypoxia, acidity, redox balance, or immunosuppressive metabolites, but current evidence often centers on metabolic disruption rather than direct immune modulation. Representative strategies target glucose and glutamine utilization, lactate transport, tryptophan metabolism, adenosine signaling, and lipid metabolism, while hypoxia, acidity, redox imbalance, and enzyme activity can serve as release triggers [101,102,103,104,105]. Because stromal and metabolic pathways are shared among malignant, immune, stromal, and normal cells, functional assessment should include cell-resolved target engagement, temporal pharmacodynamics, individual-cargo contribution, and preservation of intended combination ratios. Figure 3 summarizes nano-enabled therapeutic modalities and supporting strategies in PDAC; Table 2 compares representative nanoimmunotherapy platforms by delivery barriers, functional-delivery evidence, and pharmaceutical development limitations.

## 6. Pharmaceutical Development and Translation

### 6.1. Preclinical Models, Pharmacokinetics, and Biodistribution

**Figure 4:**
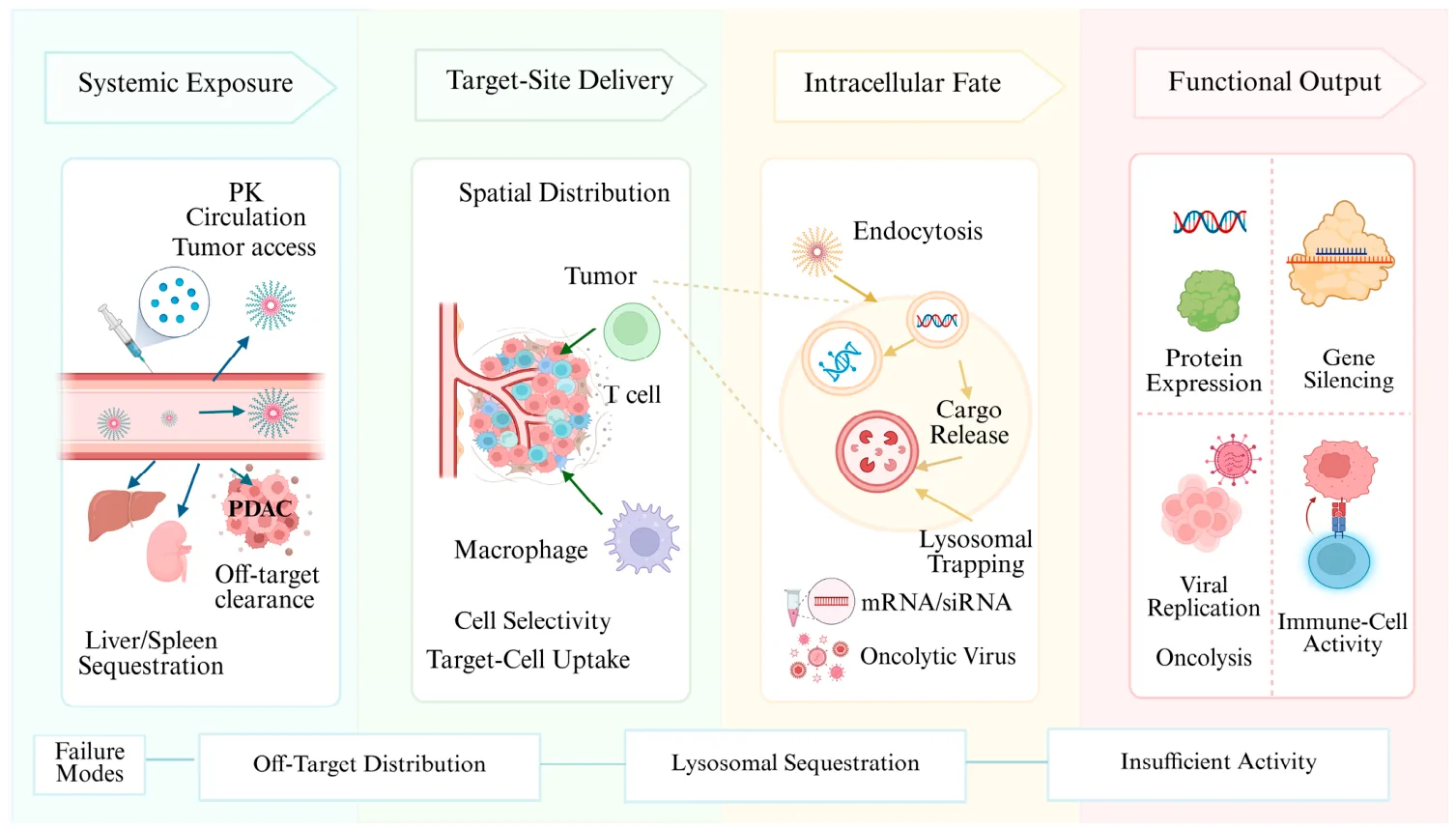
Sequential determinants of functional delivery in PDAC nanoimmunotherapy. Arrows indicate the directional progression of nanocarrier or cargo disposition, target-site delivery, and intracellular trafficking toward functional or nonproductive outcomes.

Preclinical models should reproduce the barriers relevant to the proposed delivery mechanism. Two-dimensional cultures are useful for assessing receptor dependence, cytotoxicity, transfection, and intracellular cargo activity but do not reproduce vascular transport, extracellular-matrix resistance, protein-corona formation, or multicellular immune interactions. Subcutaneous xenografts enable rapid efficacy screening but poorly reproduce the native pancreatic microenvironment, including dense desmoplastic stroma, vascular compression, immune interactions, and metastatic behavior. Xenografts in immunodeficient hosts are further limited for evaluating immune-dependent nanoimmunotherapies. Spheroids and organoids better model tissue architecture, whereas orthotopic and genetically engineered models provide greater relevance to stromal organization, perfusion, metastasis, and systemic disposition [106,107]. Immunocompetent models are essential when efficacy depends on vaccination, checkpoint modulation, macrophage reprogramming, or immune-cell engineering [5,6]. Patient-derived organoids and tumor specimens can capture target expression and interpatient heterogeneity [108,109]. Pharmacokinetic and biodistribution studies should distinguish administered dose from biologically available cargo. Plasma measurements should separate carrier-associated, intact, and released cargo when feasible, while tumor exposure should be evaluated together with hepatic and splenic accumulation and resolved spatially relative to perfused vessels, stromal regions, and malignant-cell-rich compartments [7]. Model selection and disposition analysis should therefore match the intended site and mechanism of action. Figure 4 summarizes the relationship between systemic exposure, target-site delivery, intracellular cargo fate, and mechanism-linked output. Representative analytical approaches and their interpretive limitations are summarized in Table 3.

### 6.2. Reporting and Reproducibility

Reliable comparison requires standardized reporting of formulation, model, dose, and analytical conditions. The MIRIBEL framework emphasizes material characterization, biological characterization, and experimental procedures, while animal studies should follow ARRIVE 2.0 recommendations for design and reporting [42,110]. At minimum, studies should report formulation composition, particle-size distribution, surface properties, cargo identity and integrity, loading or encapsulation efficiency, free cargo, stability, biological model, immune status, administration route, dose, dosing schedule, sampling time, analytical method, and formulation batch. Nanoparticle dose and cargo dose should be reported separately, together with clearly defined measures of delivered cargo, cellular uptake, and functional activity where applicable.

### 6.3. Critical Quality Attributes and Mechanism-Based Potency

A clinically developable nanoimmunotherapy must be defined as a reproducible drug product with controlled identity, strength, stability, safety, and biological activity [111]. Core critical quality attributes include composition, cargo identity and integrity, particle-size distribution, morphology, surface charge, encapsulation efficiency, free cargo, release characteristics, and stability. Platform-specific attributes are also required. Antibody-targeted systems should control ligand density, accessibility, and retained binding activity [61]; mRNA formulations require control of RNA integrity, capping, polyadenylation, impurities, lipid integrity, encapsulation, and expression activity [44,46,112]. Potency assays should measure biological functions causally linked to the therapeutic mechanism. Relevant endpoints include antigen expression and immune activation for mRNA vaccines, target suppression for siRNA, receptor-dependent activity for antibody-targeted systems, CAR expression and antigen-specific killing for in vivo immune-cell programming, and infectivity and oncolysis for viral formulations [47,48,61,63,86,87,88]. Potency assays should be dose-responsive, reproducible, stability-indicating, and applicable to batch release and process comparability.

### 6.4. Manufacturing, Scale-Up, Stability, and Comparability

Manufacturing variables can alter particle formation, cargo encapsulation, internal structure, and biological performance. For lipid and polymeric systems, component concentration, mixing conditions, temperature, solvent environment, and process parameters require control; microfluidic production depends on flow-rate ratio, total flow rate, pressure, and device characteristics [113]. Purification, buffer exchange, filtration, sterile processing, and filling may modify product quality [114]. Targeted formulations introduce variability through ligand conjugation, while nucleic-acid products require control of both molecular and colloidal stability [44,61,112]. Stability studies should address storage, temperature excursions, freeze–thaw exposure, light, dilution, device compatibility, and in-use conditions. Extracellular-vesicle products require standardized source-cell banking, production, purification, cargo loading, and storage [56,115]. Process comparability should not be inferred from similar mean particle size alone. Changes in scale, equipment, raw materials, conjugation chemistry, purification, storage, or manufacturing site should be evaluated using composition, cargo integrity, release, ligand function, potency, stability, and, when necessary, pharmacokinetic and biodistribution comparability. In vivo immune-cell programming likewise requires reproducible control of RNA integrity, nanoparticle composition, selective transfection, and expression kinetics [61,86,87,88]. Multiple independent batches should demonstrate consistent pharmaceutical and biological performance.

### 6.5. Biosafety and Repeat-Dose Performance

Safety assessment should consider the carrier, cargo, targeting component, administration route, immune mechanism, and dosing schedule as an integrated product. Because many nanocarriers accumulate in the liver and spleen, toxicological findings should be interpreted in relation to both cargo dose and total nanoparticle exposure [7]. Core evaluations should include hematology, serum chemistry, cytokines, complement activation, coagulation, and histopathology, guided by the observed biodistribution profile. Platform-specific risks should also be considered. Ionizable lipids can influence endosomal escape, hepatic exposure, innate immune activation, and tolerability [44,116]. Inorganic particles require assessment of dissolution, clearance, long-term retention, and inflammation [51], whereas polymeric and hybrid systems require control of residual monomers, solvents, degradation products, and membrane-disruptive activity [7,49]. Viral carriers require evaluation of tropism, replication, dissemination, shedding, and post-release infectivity [47], while antibody-targeted systems require control of free antibody, premature release, and off-target receptor engagement [61]. Protein adsorption and opsonization can increase phagocytic recognition and macrophage uptake, thereby altering systemic clearance and tissue biodistribution [117]. Together with treatment-induced immune responses, these processes may modify nanoparticle disposition during repeat administration; for example, anti-PEG antibodies can promote complement activation and accelerated clearance [41]. Repeat-dose studies should compare pharmacokinetics, organ distribution, target-site delivery, pharmacodynamic activity, and tolerability across administrations while monitoring infusion reactions, cytokine release, complement activation, anti-carrier immunity, particle disruption, and cargo leakage [41,118]. Continued development should require preservation of functional activity without progressive immunotoxicity or clinically relevant changes in disposition.

## 7. Clinical Translation and Biomarker-Guided Development

### 7.1. Clinical Precedents

Clinical experience with nanomedicine in PDAC has centered on nanoparticle albumin-bound paclitaxel and liposomal irinotecan. Although not nanoimmunotherapies, these formulations illustrate how formulation can alter drug disposition, tolerability, and combination feasibility. In the MPACT trial, gemcitabine plus nanoparticle albumin-bound paclitaxel improved overall survival compared with gemcitabine alone [4]. In NAPOLI-1, liposomal irinotecan plus 5-fluorouracil/leucovorin improved overall survival compared with 5-fluorouracil/leucovorin, while NALIRIFOX improved overall survival compared with gemcitabine plus nanoparticle albumin-bound paclitaxel in NAPOLI 3 [119,120]. These outcomes support the clinical value of formulation-dependent pharmacokinetics but do not establish uniform tumor penetration or passive nanoparticle targeting. Nanoimmunotherapies additionally require evidence of cargo stability, target-cell delivery, pharmacodynamic activity, immunogenicity, repeat-dose performance, and product consistency [7,44]. Clinical experience with nanochemotherapy should therefore not be directly extrapolated to immune-directed nanomedicines.

### 7.2. Investigational Platforms

Investigational nano-enabled approaches in PDAC include personalized vaccines, nucleic-acid delivery systems, extracellular vesicles, locally administered particulate formulations, and inorganic nanoparticles. These platforms differ in cargo, mechanism, biodistribution, and pharmaceutical requirements and should not be considered a single therapeutic class. Autogene cevumeran has been evaluated in patients with resected PDAC. This personalized lipoplex mRNA vaccine induced durable neoantigen-specific T-cell responses in a subset of patients [77,78], although clinical efficacy remains unconfirmed. Extracellular-vesicle delivery of KRASG12D-targeting siRNA has entered phase I evaluation [97], while LNP-based KRAS vaccination and inorganic platforms represent other investigational delivery strategies [52,53,73]. Early trials should link formulation disposition to cargo-specific pharmacodynamic activity.

### 7.3. Biomarker-Guided Trial Design

Patient selection for nano-enabled therapies should extend beyond PDAC diagnosis to delivery-relevant biomarkers. Perfusion, vascular accessibility, stromal architecture, macrophage abundance, target expression, and lesion location can influence delivery. Functional imaging may provide lesion-level information on nanoparticle access; for example, ferumoxytol-enhanced MRI has been explored as a predictor of nanoliposomal irinotecan uptake and response [121]. Such measurements should be interpreted as biomarkers of lesion accessibility [7,8,40]. Biomarker assessment should distinguish anatomical markers of lesion access, cellular or molecular markers of target availability, and pharmacodynamic markers of cargo activity. Vascular and macrophage density may inform nanomedicine accessibility [109], whereas receptor expression should be assessed in clinically relevant lesions because primary and metastatic tumors may differ in accessibility and target abundance. Baseline immune status and prior nanocarrier exposure may further influence clearance and immune compatibility [41]. Integrating tissue analysis, functional imaging, pharmacokinetics, and mechanism-specific pharmacodynamics can link these biomarker levels to formulation disposition and response.

### 7.4. Qualitative Stage-Gating Framework for Translational Evaluation

**Figure 5:**
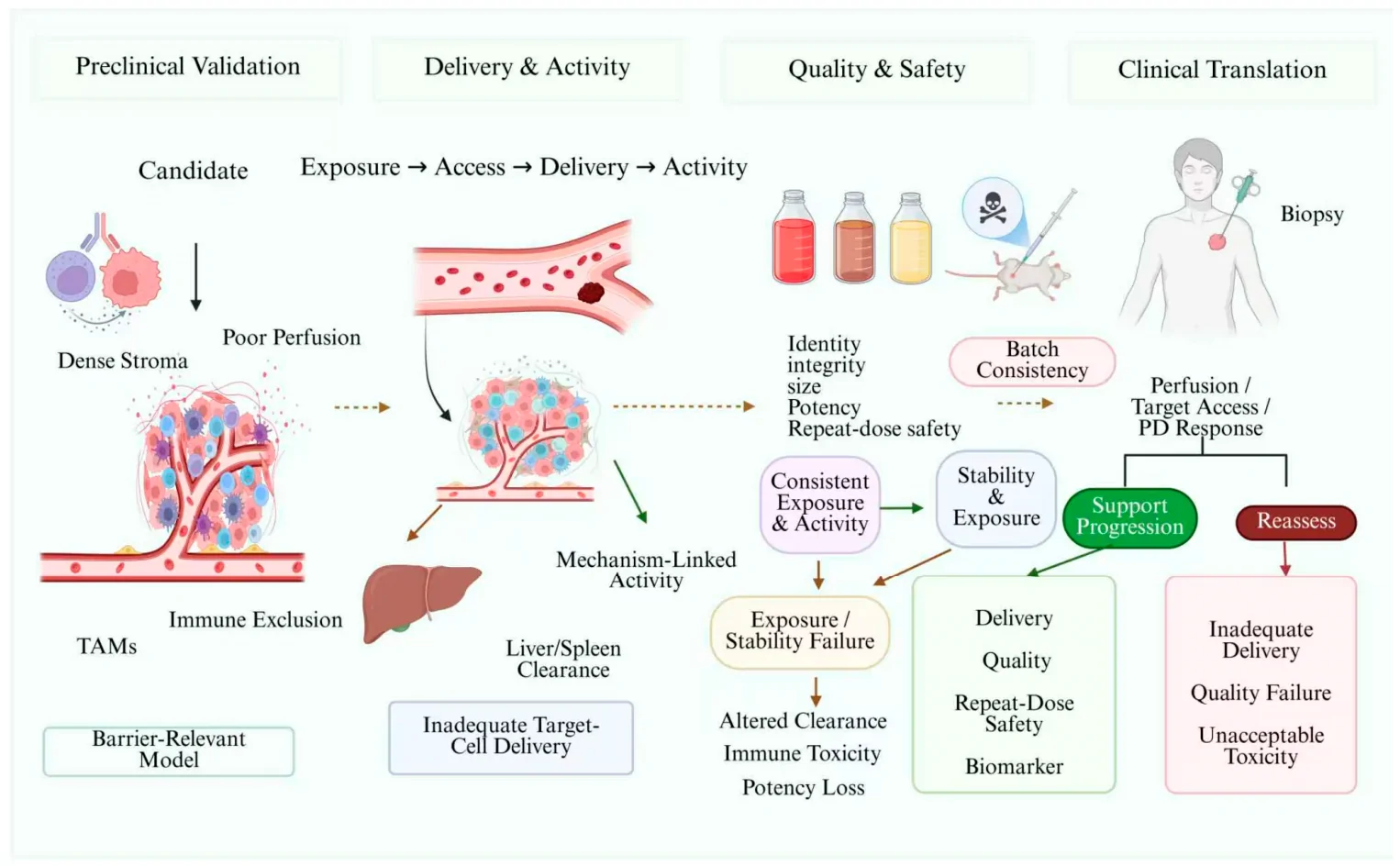
A barrier-informed qualitative stage-gating framework for PDAC nanoimmunotherapy development. Arrows indicate directional relationships and decision pathways within the framework; green arrows indicate progression-supporting outcomes, whereas brown/red arrows indicate failure or reassessment pathways, and dashed arrows indicate transitions between major development stages.

Because nanoimmunotherapy platforms differ in cargo, carrier composition, administration route, mechanism of action, and analytical readouts, universal quantitative thresholds are unlikely to apply across platforms. The proposed framework is therefore a qualitative stage-gating checklist rather than a prescriptive quantitative decision algorithm. At each stage, progression should be supported by concordant evidence relevant to the proposed mechanism, including efficacy in appropriate models and, where applicable, comparison with free cargo and matched non-targeted or inactive controls. Pharmacokinetic and biodistribution studies should establish target-site and target-cell exposure, while functional delivery should be confirmed using cargo-specific pharmacodynamic endpoints. Progression should also require consistent product quality and potency across independent batches, acceptable repeat-dose pharmacokinetics and safety, and at least one mechanism-relevant patient-selection or pharmacodynamic biomarker. Quantitative acceptance criteria should be defined prospectively for individual platforms based on mechanism of action, assay performance, exposure–response relationships, and intended clinical context. Failure to establish adequate exposure, target-cell delivery, mechanism-linked activity, product consistency, or repeat-dose performance should prompt stage-specific reassessment and, where unresolved, may preclude progression [41,111,112,114]. Figure 5 presents this barrier-informed stage-gating framework, and Table 4 summarizes the corresponding pharmaceutical and translational evaluation domains.

## 8. Future Perspectives

Future development should account for patient- and lesion-specific determinants of nanocarrier disposition, including perfusion, stromal architecture, macrophage abundance, target accessibility, prior treatment, and metastatic-site biology. Prospective studies should integrate imaging and tissue biomarkers with pharmacokinetics, spatial biodistribution, and cargo-specific pharmacodynamic endpoints [109,121]. Primary and metastatic lesions should be evaluated separately when their delivery barriers differ. Patient-derived biological media may improve prediction of protein-corona effects, anti-carrier immunity, and repeat-dose disposition [40,41]. Platform complexity should be justified by a clear pharmacological advantage. In vivo immune-cell programming requires selective transfection, controlled expression, limited off-target activity, and mechanism-based potency assays [61,86,87,88]. For combination products, each component should address a defined resistance mechanism, and co-formulation should provide an advantage over simpler or sequential regimens.

## 9. Conclusions

The therapeutic value of nanoimmunotherapy in PDAC depends on progression from tumor access to target-cell delivery, cargo release, target engagement, and pharmacological activity. A barrier-informed pharmaceutics framework integrates biological access with formulation design, pharmacokinetics, biodistribution, potency, product quality, safety, and biomarker-guided translation. Successful development requires concordant evidence of functional delivery, reproducible product performance, repeat-dose feasibility, and clinically relevant patient selection.

## Figures and Tables

**Table 1:** Barrier-to-formulation design map for PDAC nanoimmunotherapy.

PDAC-Associated Barrier	Consequence for Drug Delivery	Formulation Implication	Recommended Readout	Potentially Misleading Surrogate	Ref.
Vascular compression and heterogeneous perfusion	Limited tumor entry and spatially heterogeneous exposure	Optimize route, systemic disposition, and physicochemical properties for lesion access rather than circulation alone	Lesion-level exposure; vascular proximity; spatial distribution	Plasma half-life; bulk tumor concentration	[15,16]
Dense stroma and CAF heterogeneity	Restricted extravasation and penetration into malignant-cell-rich regions	Favor penetration-enhancing or selective stromal-normalizing strategies; avoid indiscriminate stromal depletion	Penetration relative to perfused vessels and stromal architecture	Total tumor fluorescence	[17,23]
Protein corona and phagocytic sequestration	Altered surface identity, ligand masking, and hepatic/splenic or macrophage uptake	Control surface chemistry and biological identity; assess cell-specific disposition	Corona composition; liver/spleen exposure; target-cell uptake	In vitro receptor binding or total particle uptake	[7,40,43]
Endosomal or lysosomal sequestration	Cellular uptake without productive intracellular delivery	Incorporate cargo-appropriate release and endosomal-escape mechanisms	Cytosolic cargo availability; protein expression; gene silencing	Cellular internalization alone	[40,44]
Immune exclusion and suppressive myeloid populations	Reduced immune activation despite successful cargo delivery	Match target-cell selection and immunomodulatory strategy to the dominant immune barrier	Antigen presentation; T-cell activation; immune-cell reprogramming	Bulk immune-cell or cytokine changes	[19,25]
Hypoxia, acidosis, and metabolic heterogeneity	Variable trigger activation and risk of activity in unintended cell populations	Calibrate responsive release thresholds and incorporate cell-type selectivity	Trigger-dependent release; cell-resolved target engagement	Release under uniform in vitro conditions	[31,33,45]
Anti-carrier immunity during repeated dosing	Accelerated clearance, altered biodistribution, and loss of functional delivery	Evaluate surface strategy and formulation performance across repeated administrations	Anti-carrier antibodies; repeat-dose PK/PD; cargo activity	First-dose pharmacokinetics	[41]

**Table 2:** Representative PDAC nanoimmunotherapy platforms: delivery barriers, functional outcomes, and development limitations.

Nanoimmunotherapy Platform	Representative Formulation and Cargo	Administration and Experimental Model	Dominant Delivery Barrier/Target Compartment	Evidence Supporting Functional Delivery	Translational Stage and Key Pharmaceutical Challenges	Ref.
Nanoengineered oncolytic-virus delivery	AdNuPARmE1A coated with PEGylated oligopeptide-modified poly(β-amino ester)s	Intravenous administration; PDAC mouse models with pre-existing anti-adenovirus antibodies	Viral neutralization, hepatic sequestration, and insufficient tumor exposure	Reduced hepatic sequestration, prolonged circulation, preserved viral infectivity, and maintained antitumor activity	Preclinical proof-of-concept; formulation composition, post-release infectivity, immunogenicity, and scalable manufacturing require further optimization	[48]
Pancreas-directed mRNA delivery using ionizable lipid nanoparticles	Arginine/histidine-modified ionizable AH-LNP carrying therapeutic mRNA	Intraperitoneal administration; pancreatic cancer models and non-human primates	Limited pancreatic access and inefficient organ-selective transfection	Pancreatic accumulation and VLDLR-dependent uptake enabled local cytokine expression and enhanced vaccine/CAR-T responses	Advanced preclinical; translation requires validation of route feasibility and relevance to human pancreatic anatomy	[71]
Nanovaccine combined with immunogenic cell death induction	Spleen-targeted LNP carrying KRAS^G12D^ mRNA and 3M-052 combined with irinotecan silicasomes	Intravenous administration; orthotopic KPC PDAC model	Insufficient antigen presentation and limited tumor-antigen release	KRAS-specific T-cell priming, tumor-localized immunogenic cell death, and improved antitumor response	Preclinical proof-of-concept; dual-platform manufacturing, dose optimization, and contribution of individual components require clarification	[73]
Personalized neoantigen nanovaccination	Autogene cevumeran containing patient-specific neoantigen mRNAs formulated in lipoplex nanoparticles	Intravenous administration; phase I adjuvant study in resected PDAC	Antigen heterogeneity and limited endogenous T-cell priming	Induction of neoantigen-specific immune responses, including durable polyfunctional CD8^+^ T-cell responses	Early clinical investigation (Phase I); individualized manufacturing, limited cohort size, and clinical efficacy validation remain important considerations	[77,78]
Nanoparticle-assisted in vivo T-cell programming	Anti-CD5-targeted LNP carrying FAP-CAR mRNA	Intravenous administration; syngeneic PDAC model	FAP-positive stromal barriers, immune exclusion, and limitations of ex vivo cell manufacturing	Transient CAR expression in circulating, splenic, and tumor-infiltrating T cells with stromal remodeling and tumor control	Preclinical proof-of-concept; redosing strategy, off-target transfection, expression duration, and systemic safety require evaluation	[86]
Nanoparticle-assisted macrophage programming	Macrophage-targeted PSβ-LNP carrying CAR mRNA	Intraperitoneal administration; PAN02 peritoneal and orthotopic PDAC models	Immunosuppressive macrophage populations and peritoneal tumor dissemination	Preferential macrophage transfection, proinflammatory CAR activity, adaptive immune activation, and enhanced checkpoint response	Preclinical proof-of-concept; biodistribution, cell selectivity, expression kinetics, and inflammatory toxicity require optimization	[87]
Nano-enabled bacterial immunomodulation	Engineered E. coli Nissle carrying collagenase- and anti-PD-L1-containing protein cages	Intravenous administration; orthotopic KPC PDAC model	Collagen-rich stroma, poor penetration, and PD-L1-mediated immune suppression	Tumor-selective localization and ROS-triggered protein cage release enhanced stromal remodeling and checkpoint activity	Preclinical proof-of-concept; living-product safety, localization control, tumor heterogeneity, and manufacturing complexity remain challenges	[91]
Extracellular-vesicle-mediated RNA interference	Mesenchymal-cell-derived iExosomes carrying KRAS^G12D^-targeting siRNA or shRNA	Intraperitoneal administration; orthotopic, genetically engineered, and metastatic PDAC models	RNA instability, phagocytic clearance, and selective delivery to KRAS-mutant cells	CD47-dependent persistence and macropinocytic uptake reduced KRAS^G12D^ expression, tumor burden, and metastasis	Preclinical proof-of-concept; source-cell control, cargo loading, purification, dose normalization, batch consistency, biodistribution, and long-term safety require standardization	[97]

**Table 3:** Representative methods for assessing functional delivery of nanoimmunotherapies.

Method	Information Provided	Major Limitation	Interpretation
Fluorescence imaging	Spatial localization of labeled carrier or cargo	Signal may persist after carrier disassembly or cargo degradation	Localization alone does not demonstrate functional cargo delivery
Radiolabeling (PET/SPECT)	Quantitative PK and biodistribution	Label detachment or metabolism may uncouple signal from intact material	Tumor radioactivity indicates exposure, not target-cell delivery
Flow cytometry	Cell-specific association or uptake	Surface-bound and internalized signals may be difficult to distinguish	Supports cell-resolved uptake when appropriate controls are used
Mass spectrometry	Molecularly specific cargo or metabolite quantification	Limited cell-specific information in bulk tissue analysis	Demonstrates molecular exposure but not necessarily intracellular function
Subcellular fractionation	Intracellular compartmental distribution	Cross-contamination between fractions	Supports intracellular availability when fraction purity is validated
Spatial transcriptomics	Spatially resolved pharmacodynamic responses	Indirect and does not directly detect carrier or cargo	Supports mechanism-linked biological activity rather than direct delivery

**Table 4:** Pharmaceutical and translational evaluation framework for PDAC nanoimmunotherapies.

Development Domain	Core Requirement	Recommended Measurements	Stage-Gating Consideration	Key References
Product identity and critical quality attributes	Define a reproducible carrier–cargo product with controlled identity, strength, and quality	Composition; cargo identity/integrity; size distribution; PDI; morphology; surface charge; encapsulation; free cargo; ligand density/accessibility; release; sterility	Product-defining attributes remain within justified ranges across independent batches	[111]
Stability and manufacturability	Preserve molecular, colloidal, and functional integrity during manufacture, storage, preparation, and administration	Process parameters; raw-material variability; degradation; aggregation; leakage; freeze–thaw tolerance; lyophilization; reconstitution; device compatibility; post-storage potency	Intended-scale batches retain CQAs and potency; process changes are supported by comparability studies	[112,114]
Biological identity and functional delivery	Distinguish particle accumulation and cellular uptake from productive cargo delivery	Protein corona; carrier-associated, intact, and released cargo; target-cell uptake; intracellular trafficking; endosomal escape; cargo availability; mechanism-specific activity	The formulation reaches the intended cell and pharmacological compartment and produces the required functional response	[40,43]
Preclinical model relevance	Use models that reproduce the barriers relevant to the delivery mechanism	Tumor location; immune status; stroma; perfusion; target expression; metastatic distribution; target-cell identity; pharmacodynamic response	Efficacy is reproduced in a model containing the relevant delivery and immune barriers	[106,107]
Pharmacokinetics and biodistribution	Link administered dose to target-site and target-cell exposure	Plasma PK; carrier-associated/intact/released cargo; liver/spleen exposure; spatial penetration; vascular proximity; cell-specific uptake; lesion distribution; repeat-dose PK	Adequate target-site and target-cell exposure is achieved without excessive off-target accumulation	[7,109]
Mechanism-based potency	Measure biological activity causally linked to the proposed mechanism	mRNA expression; siRNA target suppression; receptor-dependent activity; CAR expression and killing; viral infectivity and oncolysis	Potency is dose-responsive, reproducible, stability-indicating, and suitable for batch release and comparability	[46,47,61,86]
Reporting quality and reproducibility	Report sufficient information to identify the product and reproduce the study	Material/biological characterization; cargo and nanoparticle doses; route; schedule; sampling; analytical method; batch; sample size; randomization; blinding	Administered dose, delivered dose, cellular uptake, and functional activity can be clearly distinguished and reproduced	[42,110]
Immunotoxicity and repeat-dose performance	Establish immune compatibility and safety throughout the intended regimen	Complement; cytokines; anti-carrier antibodies; infusion reactions; accelerated clearance; particle disruption; cargo leakage; histopathology; repeat-dose PD	Repeated dosing does not cause unacceptable clearance, activity loss, progressive immunotoxicity, or altered organ distribution	[41,118]
Biomarker-guided translation	Identify patients and lesions capable of access, target-cell delivery, and functional response	Perfusion; vascular/macrophage density; stromal organization; target accessibility; imaging/tissue biomarkers; exposure-linked PD	A prospectively testable selection or pharmacodynamic biomarker is linked to the delivery mechanism	[109,121]
Integrated stage-gating assessment	Align exposure, functional delivery, product quality, safety, and clinical feasibility	Appropriate controls; durable efficacy; batch consistency; repeat-dose exposure; pharmacodynamics; manufacturability; feasible administration	Progression should be supported by concordant evidence of exposure, target-cell activity, product consistency, repeat-dose performance, and biomarker support	[7,111]

## Data Availability

No new data were created or analyzed in this study. Data sharing is not applicable to this article.

## Supplementary Materials

The following supporting information can be downloaded at: https://www.mdpi.com/article/10.3390/pharmaceutics18101305/s1. Table S1: Literature search strategy and evidence-selection process; Table S2: Publications identified through supplementary evidence selection and rationale for inclusion.

## Abbreviations

The following abbreviations are used in this manuscript:

PDAC	Pancreatic ductal adenocarcinoma
CAF	Cancer-associated fibroblast
TAM	Tumor-associated macrophage
TME	Tumor microenvironment
LNP	Lipid nanoparticle
mRNA	Messenger RNA
siRNA	Small interfering RNA
CAR	Chimeric antigen receptor
CAR-T	Chimeric antigen receptor T cell
ICI	Immune checkpoint inhibitor
PD-1	Programmed cell death protein 1
PD-L1	Programmed death-ligand 1
PK	Pharmacokinetics
PD	Pharmacodynamics
CQA	Critical quality attribute
PEG	Polyethylene glycol
ROS	Reactive oxygen species
ECM	Extracellular matrix
MDSC	Myeloid-derived suppressor cell
NK	Natural killer cell
IDO1	Indoleamine 2,3-dioxygenase 1
TGF-β	Transforming growth factor beta
CXCR4	C-X-C chemokine receptor type 4
