# Supplementary material for: Functional Delivery of Nanoimmunotherapies in Pancreatic Ductal Adenocarcinoma: A Barrier-Informed Pharmaceutics Framework
Source: Pharmaceutics. 2026 Oct 15;18(10):1305. doi: 10.3390/pharmaceutics18101305 (PMC13708326; doi:10.3390/pharmaceutics18101305)
Supplement: Supplementary file 1 [file pharmaceutics-18-01305-s001.zip › pharmaceutics-4528610-supplementary.pdf]

Table S1. Literature search strategy and evidence selection process

Item	Description
Review type	Critical narrative review informed by a structured literature search
Databases	PubMed and Web of Science
Search period	Publications available up to 31 August 2026
Search approach	Multiple complementary searches were performed using combinations of terms related to PDAC, biological barriers, nanomedicine and drug delivery, immunotherapy, extracellular vesicles, nucleic-acid therapeutics, oncolytic viruses, immune-cell engineering, and pharmaceutical or translational considerations.
PDAC terms	"pancreatic ductal adenocarcinoma" OR "pancreatic cancer" OR PDAC
Biological-barrier terms	"tumor microenvironment" OR stroma* OR desmoplas* OR fibroblast* OR macrophage* OR myeloid* OR immunosuppress* OR hypoxi* OR metabol* OR hyaluronan
Nanomedicine/delivery terms	nanoparticl* OR nanomedicin* OR nanocarrier* OR "drug delivery" OR "lipid nanoparticle*" OR LNP OR liposom* OR exosom* OR "extracellular vesicle*"
Immunotherapy terms	immunotherap* OR immunomodulat* OR vaccin* OR checkpoint* OR PD-1 OR PD-L1 OR CTLA-4 OR STING OR TLR* OR "CAR-T" OR "CAR macrophage*"
Cargo/platform terms	mRNA OR siRNA OR nucleic acid* OR KRAS OR "oncolytic virus*" OR virotherap* OR "neoantigen vaccine*" OR "RNA vaccine*"
Representative search structure	Representative combinations included: (PDAC terms) AND (biological-barrier terms); (PDAC terms) AND (nanomedicine/delivery terms) AND (immunotherapy terms); and (PDAC terms) AND [(nanomedicine/delivery terms AND immunotherapy-related terms) OR (cargo/platform terms)]. Search syntax and field tags were adapted as appropriate for PubMed and Web of Science.
Availability of original search documentation	The retained search documentation supports the principal search concepts, Boolean combinations, and PubMed-formatted search strategies. However, the original database-specific and query-level hit counts and the exact Web of Science-formatted query-history strings were not prospectively archived. These unavailable historical data were therefore not retrospectively estimated or reconstructed.
Records in the consolidated	7,341

Item	Description
bibliographic dataset	
Deduplication	Records retrieved through the complementary searches were consolidated into a working bibliographic dataset and deduplicated primarily using normalized publication titles.
Unique records after deduplication	6,383
Title-level relevance screening	Titles were screened broadly for relevance to PDAC, biological barriers, nano-enabled or immune-directed therapy, drug delivery, or directly applicable pharmaceutical and translational principles. Records clearly outside these domains were excluded.
Records retained after title-level relevance screening	5,148
Framework-relevance assessment	The retained records were further assessed for potential contribution to the barrier-informed pharmaceutics framework, including biological barriers, formulation and delivery design, nano-enabled immune intervention, functional delivery, pharmacokinetics and biodistribution, intracellular trafficking, pharmaceutical quality, manufacturing and stability, biosafety and repeat-dose performance, and biomarker-guided translation.
Potentially framework-relevant records	1,175
Narrative evidence selection	Because this was a critical narrative review rather than a systematic review, publications from the framework-relevant pool were selected for citation according to their direct relevance to the mechanistic, pharmaceutical, and translational arguments developed in the manuscript. Evidence selection was therefore interpretive rather than based on quantitative meta-analytic eligibility criteria.
Database-derived publications cited	69
Supplementary evidence selection	Additional publications were identified manually when needed to support foundational concepts, methodological considerations, clinical background, or broadly applicable pharmaceutical and translational principles that were not adequately represented in the database-derived evidence pool.
Supplementary publications cited	52
Total publications cited	121

Note: The numerical workflow reported in this table (7,341 consolidated bibliographic records → 6,383 unique records after deduplication → 5,148 records retained after title-level relevance screening → 1,175 potentially framework-relevant records) is based on the retained bibliographic and screening records and reflects the evidence-selection process used for this review. The original database-specific and query-level hit counts generated when the individual searches were conducted were not prospectively archived and therefore cannot be reported retrospectively. Similarly, the exact Web of Science-formatted query-history strings were not retained. To avoid introducing unverifiable historical information, these unavailable data were not retrospectively estimated or reconstructed. Because this was a critical narrative review rather than a systematic review, final selection of publications from the framework-relevant pool was based on their relevance to the predefined mechanistic, pharmaceutical, and translational themes of the review.

Table S2. Publications identified through supplementary evidence selection and rationale for inclusion

Ref.	Publication	Evidencecategory		Whynotrepresentedindatabase
2	(Rahib et al., 2014), *Projecting cancer incidence and deaths to 2030: the unexpected burden of thyroid, liver, and pancreas cancers in the United States*	Clinical background	Provides epidemiological context for the clinical burden of pancreatic cancer.	Background epidemiology was not a principal target of the structured nanoimmunotherapy searches.
7	(Mitchell et al., 2021), *Engineering precision nanoparticles for drug delivery*	General nanomedicine	Provides foundational principles of nanoparticle design, biological delivery, and translation.	Broad nanomedicine literature not restricted to PDAC.
8	(Rosenblum et al., 2018), *Progress and challenges towards targeted delivery of cancer therapeutics*	Drug-delivery principles	Supports general principles and limitations of targeted therapeutic delivery.	Broad cancer-delivery literature not restricted to PDAC.
14	(Yang et al., 2023), *Cancer-associated fibroblasts: from basic science to anticancer therapy*	Tumor-stroma biology	Provides broader biological context for CAF heterogeneity and stromal functions relevant to barrier-informed delivery.	Broad CAF literature not restricted to PDAC or nanoimmunotherapy.
23	(Özdemir et al., 2014), *Depletion of carcinoma-associated fibroblasts and fibrosis induces immunosuppression and accelerates pancreas cancer with reduced survival*	Stromal biology	Supports the caution that indiscriminate stromal depletion can worsen immunosuppression and disease outcome.	Included as mechanistic stromal context rather than as core nanoimmunotherapy evidence.
26	(Noman et al., 2014), *PD-L1 is a novel direct target of HIF-1α, and its blockade under hypoxia enhanced MDSC-mediated T cell activation*	Hypoxia/immunology	Supports the mechanistic link between hypoxia, HIF-1α, PD-L1 regulation, and immune suppression.	General tumor-immunology evidence not restricted to PDAC or nanomedicine.
27	(Sethumadhavan et al., 2017), *Hypoxia and hypoxia-inducible factor (HIF) downregulate antigen-presenting MHC class I molecules limiting tumor cell recognition by T cells*	Hypoxia/immunology	Supports hypoxia-mediated impairment of antigen presentation and T-cell recognition.	General mechanistic immunology evidence outside the PDAC nanoimmunotherapy focus.
28	(Zeng et al., 2025), *Advances in the mechanisms of HIF-1α-enhanced tumor glycolysis and its relation to dedifferentiation*	Metabolic biology	Provides mechanistic context for HIF-1α-driven glycolytic reprogramming relevant to hypoxic tumor barriers.	Broad mechanistic literature not restricted to PDAC or nanomedicine.
40	(Voke et al., 2025), *Protein corona*	Biological identity	Supports the effect of	General LNP study without a

Ref.	Publication	Evidencecategory		Whynotrepresentedindatabase
	formed on lipid nanoparticles compromises delivery efficiency of mRNA cargo		protein-corona formation on LNP-mediated functional cargo delivery.	PDAC focus.
41	(Estapé Senti et al., 2022), *Anti-PEG antibodies compromise the integrity of PEGylated lipid-based nanoparticles via complement*	Repeat-dose safety	Supports anti-PEG antibodies, complement activation, and nanoparticle-integrity considerations.	General nanomedicine safety evidence.
42	(Faria et al., 2018), *Minimum information reporting in bio–nano experimental literature*	Methodological/reporting standard	Supports standardized reporting of bio–nano experimental studies.	Methodological literature without a PDAC focus.
43	(Monopoli et al., 2012), *Biomolecular coronas provide the biological identity of nanosized materials*	Biological identity	Provides the foundational concept of the biomolecular corona as the biological identity of nanomaterials.	Foundational nanoscience literature without a PDAC focus.
44	(Hou et al., 2021), *Lipid nanoparticles for mRNA delivery*	LNP delivery	Provides general principles of LNP composition and mRNA delivery.	Broad LNP literature not restricted to PDAC.
45	(Mura et al., 2013), *Stimuli-responsive nanocarriers for drug delivery*	Formulation design	Supports general principles of stimuli-responsive nanocarriers.	General drug-delivery literature not restricted to PDAC.
46	(Kulkarni et al., 2021), *The current landscape of nucleic acid therapeutics*	Nucleic-acid therapeutics	Provides broader context for nucleic-acid therapeutics and their delivery requirements.	Broad nucleic-acid therapeutics literature not restricted to PDAC.
47	(Ma et al., 2023), oncolytic virus-based cancer immunotherapy	Oncolytic-virus platform	Provides general mechanistic and translational context for oncolytic-virus immunotherapy.	Broad cancer-immunotherapy literature rather than PDAC-specific nanoimmunotherapy evidence.
49	(Yu et al., 2023), *Smart Polymeric Nanoparticles in Cancer Immunotherapy*	Nanoimmunotherapy	Supports general polymeric nanoparticle design principles for cancer immunotherapy.	Not PDAC-specific.
50	(Tang et al., 2022), *Multifunctional inorganic nanomaterials for cancer photoimmunotherapy*	Nanoimmunotherapy	Provides general evidence on inorganic nanomaterials and photoimmunotherapy.	Not PDAC-specific.
51	(Singh et al., 2025), *Advanced*	Nanomaterial platform	Provides general	Broad nanomedicine literature

Ref.	Publication	Evidencecategory		Whynotrepresentedindatabase
	Nanomaterials for Cancer Therapy: Gold, Silver, and Iron Oxide Nanoparticles in Oncological Applications		platform information for inorganic nanoparticles used in oncology.	not restricted to PDAC.
53	(Meng et al., 2025), *Inorganic Nanobiomaterials Boost Tumor Immunotherapy: Strategies and Applications*	Nanoimmunotherapy	Supports general inorganic nanobiomaterial strategies for tumor immunotherapy.	Not PDAC-specific.
54	(Cheng et al., 2021), *Nanomaterials for cancer therapy: current progress and perspectives*	Nanomaterial platform	Provides general principles of nanomaterial-based cancer therapy.	Broad oncology/nanomedicine literature.
55	(Liu et al., 2024), *Advances in Nanomaterials for Immunotherapeutic Improvement of Cancer Chemotherapy*	Nanoimmunotherapy	Supports mechanisms by which nanomaterials can enhance cancer immunotherapy.	Not PDAC-specific.
56	(Welsh et al., 2024), *Minimal information for studies of extracellular vesicles (MISEV2023): From basic to advanced approaches*	EV methodological standard	Provides consensus guidance on EV characterization, purity, normalization, and reporting.	Methodological guideline rather than a PDAC-specific therapeutic study.
57	(Zhu & Li, 2023), *Nanomaterials in tumor immunotherapy: new strategies and challenges*	Nanoimmunotherapy	Provides general context for nanomaterial-enabled tumor immunotherapy.	Not PDAC-specific.
58	(Gong et al., 2021), *Nanomaterials for T-cell cancer immunotherapy*	T-cell nanoengineering	Supports general principles of nanomaterial-assisted T-cell immunotherapy.	Not PDAC-specific.
59	(Miao et al., 2024), *Regulating protein corona on nanovesicles by glycosylated polyhydroxy polymer modification for efficient drug delivery*	Protein corona	Supports strategies for controlling biological identity and protein-corona effects.	General nanomedicine literature.
60	(Salvati et al., 2013), *Transferrin-functionalized nanoparticles lose their targeting capabilities when a biomolecule corona adsorbs on the surface*	Targeting/biological identity	Demonstrates that biomolecular-corona formation can compromise receptor-mediated nanoparticle targeting.	Foundational non-PDAC nanomedicine evidence.
61	(Wang et al., 2024), *Unleashing the Potential: Designing Antibody-Targeted Lipid Nanoparticles for Industrial Applications with CMC Considerations and Clinical Outlook*	CMC/targeted LNP	Supports formulation and CMC considerations for targeted LNP systems.	General pharmaceutical-development literature.
65	(Chen et al., 2026), *Genetic*	Oncolytic-virus	Provides platform-level	Broad oncolytic-virus platform

Ref.	Publication	Evidencecategory		Whynotrepresentedindatabase
	engineering of systemically injectable oncolytic viruses for pyroptosis-accelerated cancer virotherapy	engineering	evidence for systemic oncolytic-virus engineering and mechanism-linked pyroptotic immunotherapy.	evidence not restricted to PDAC.
66	(Wedge et al., 2022), *Virally programmed extracellular vesicles sensitize cancer cells to oncolytic virus and small molecule therapy*	Virus–EV biology	Supports mechanistic interactions between oncolytic viruses, extracellular vesicles, and therapeutic sensitization.	General platform/mechanistic evidence rather than PDAC-specific evidence.
70	(Selvanesan et al., 2022), *Listeria delivers tetanus toxoid protein to pancreatic tumors and induces cancer cell death in mice*	Engineered microbial delivery	Provides proof of concept for tumor-directed microbial delivery and immune-mediated antitumor activity in pancreatic cancer.	Engineered bacterial delivery was used as supporting context rather than core nanoimmunotherapy evidence.
72	(Melamed et al., 2023), *Ionizable lipid nanoparticles deliver mRNA to pancreatic β cells via macrophage-mediated gene transfer*	Functional delivery	Provides proof of cell-directed functional mRNA delivery by ionizable LNPs.	Non-PDAC application used to support a general delivery principle.
84	(Rurik et al., 2022), *CAR T cells produced in vivo to treat cardiac injury*	In-vivo CAR programming	Provides proof of principle for in-vivo generation of CAR-T cells using mRNA nanotechnology.	Non-PDAC/non-oncology application used as technological context.
87	(Gu et al., 2025), *Intraperitoneal programming of tailored CAR macrophages via mRNA lipid nanoparticle to boost cancer immunotherapy*	Immune-cell engineering	Supports feasibility of nanoparticle-mediated in-vivo CAR-macrophage programming.	Non-PDAC platform evidence.
88	(Billingsley et al., 2024), *In Vivo mRNA CAR T Cell Engineering via Targeted Ionizable Lipid Nanoparticles with Extrahepatic Tropism*	Immune-cell engineering	Supports targeted in-vivo CAR-T engineering using ionizable LNPs.	Non-PDAC platform evidence.
89	(Ayala Ceja et al., 2024), *CAR-T cell manufacturing: Major process parameters and next-generation strategies*	Manufacturing	Supports manufacturing considerations for engineered immune-cell products.	General manufacturing literature rather than PDAC-specific evidence.
95	(Yen et al., 2025), *Enzymatically responsive nanocarriers targeting PD-1 and TGF-β pathways reverse immunotherapeutic resistance and elicit robust therapeutic efficacy*	Combination delivery	Supports stimulus-responsive, coordinated delivery targeting complementary	Used as supplementary platform evidence; the specific historical reason it was not represented in the retained pool was not reconstructed.

Ref.	Publication	Evidencecategory		Whynotrepresentedindatabase
			immunosuppressive pathways.	
96	(Elsharkasy et al., 2020), *Extracellular vesicles as drug delivery systems: Why and how?*	EV delivery	Provides general principles and limitations of EV-based drug delivery.	Broad EV literature without a PDAC focus.
99	(M. Li et al., 2025), *J-Aggregated indocyanine green-loaded exosomes enable photoactivatable cytoplasmic delivery of STING agonist for targeted pancreatic cancer immunotherapy*	EV functional delivery	Provides a PDAC example linking engineered exosome delivery to cytoplasmic cargo release and STING-mediated immune activity.	Not represented in the retained database-derived pool; the specific historical reason was not reconstructed.
100	(Van Cutsem et al., 2020), *Randomized Phase III Trial of Pegvorhyaluronidase Alfa With Nab-Paclitaxel Plus Gemcitabine for Patients With Hyaluronan-High Metastatic Pancreatic Adenocarcinoma*	Clinical stromal modulation	Provides clinical evidence on hyaluronan-targeted stromal modulation and illustrates limitations of barrier modification alone.	Clinical stromal-modulation evidence used for translational context rather than as core nanoimmunotherapy evidence.
101	(Y. Li et al., 2025), *An 'AND' Logic Gate Nanoreactor for Metabolic Remodeling in Starvation-Ferroptosis-Immunotherapy of Pancreatic Cancer*	Metabolic nanotherapy	Provides a PDAC example of nanomaterial-enabled metabolic remodeling linked to immunotherapy.	Not represented in the retained database-derived pool; the specific historical reason was not reconstructed.
107	(Hingorani et al., 2005), *Trp53R172H and KrasG12D cooperate to promote chromosomal instability and widely metastatic pancreatic ductal adenocarcinoma in mice*	Preclinical model	Provides foundational evidence for a genetically engineered PDAC model.	Published in 2005, before the 2010 lower date limit applied in the relevant searches.
109	(May et al., 2024), *Histopathological biomarkers for predicting the tumour accumulation of nanomedicines*	Biomarker/translation	Supports tissue biomarkers of nanomedicine accumulation and lesion accessibility.	Broader translational nanomedicine evidence rather than PDAC-specific evidence.
110	(Percie du Sert et al., 2020), *The ARRIVE guidelines 2.0: Updated guidelines for reporting animal research*	Preclinical methodology	Supports rigorous design and reporting of animal experiments.	Methodological guideline, not disease-specific therapeutic evidence.
111	(de Vlieger et al., 2019), AAPS Guidance Forum report on FDA guidance for drug products containing nanomaterials	CQA/regulatory	Supports critical quality attributes and regulatory development of nanomedicines.	General pharmaceutical/regulatory literature.
112	(Schoenmaker et al., 2021), *mRNA-lipid nanoparticle COVID-19*	Stability	Supports structural and stability	General LNP pharmaceutical evidence rather than

Ref.	Publication	Evidencecategory		Whynotrepresentedindatabase
	vaccines: Structure and stability		considerations for mRNA-LNP products.	PDAC-specific evidence.
113	(Belliveau et al., 2012), *Microfluidic Synthesis of Highly Potent Limit-size Lipid Nanoparticles for In Vivo Delivery of siRNA*	Manufacturing	Supports process control and reproducible LNP manufacturing.	General manufacturing literature.
114	(Mehta et al., 2023), *Lipid-Based Nanoparticles for Drug/Gene Delivery: An Overview of the Production Techniques and Difficulties Encountered in Their Industrial Development*	Manufacturing	Supports scale-up and industrial-development considerations.	General pharmaceutical-development literature.
116	(Sabnis et al., 2018), *A Novel Amino Lipid Series for mRNA Delivery: Improved Endosomal Escape and Sustained Pharmacology and Safety in Non-human Primates*	Functional delivery/safety	Supports endosomal escape, pharmacology, and translational safety considerations.	General LNP delivery evidence rather than PDAC-specific evidence.
117	(Gustafson et al., 2015), *Nanoparticle Uptake: The Phagocyte Problem*	Biodistribution	Supports macrophage uptake and clearance as determinants of nanoparticle disposition.	General nanomedicine literature.
118	(Szebeni et al., 2018), *Roadmap and strategy for overcoming infusion reactions to nanomedicines*	Biosafety	Supports complement-mediated and infusion-related safety considerations.	General nanomedicine safety literature.
121	(Ramanathan et al., 2017), *Correlation between Ferumoxytol Uptake in Tumor Lesions by MRI and Response to Nanoliposomal Irinotecan in Patients with Advanced Solid Tumors: A Pilot Study*	Biomarker/translation	Supports imaging-based assessment of lesion accessibility and nanomedicine accumulation.	Broader solid-tumor translational study used to support a general biomarker principle.

Note: Supplementary publications were identified during narrative evidence development to support foundational, methodological, clinical-background, mechanistic, pharmaceutical, or translational aspects not adequately represented in the retained database-derived literature pool. Where the original reason that a publication was not represented in the retained pool could not be determined from the preserved search records, no retrospective cause of non-retrieval was assigned.

Ayala Ceja, M., Khericha, M., Harris, C. M., Puig-Saus, C., & Chen, Y. Y. (2024). CAR-T cell manufacturing: Major process parameters and next-generation strategies. *J Exp Med*, *221*(2). https://doi.org/10.1084/jem.20230903

Belliveau, N. M., Huft, J., Lin, P. J., Chen, S., Leung, A. K., Leaver, T. J., Wild, A. W., Lee, J. B., Taylor, R. J., Tam, Y. K., Hansen, C. L., & Cullis, P. R. (2012). Microfluidic Synthesis of Highly Potent Limit-size Lipid Nanoparticles for In Vivo Delivery of siRNA. *Mol Ther Nucleic Acids*, *1*(8), e37. https://doi.org/10.1038/mtna.2012.28

Billingsley, M. M., Gong, N., Mukalel, A. J., Thatte, A. S., El-Mayta, R., Patel, S. K., Metzloff, A. E., Swingle, K. L., Han, X., Xue, L., Hamilton, A. G., Safford, H. C., Alameh, M. G., Papp, T. E., Parhiz, H., Weissman, D., & Mitchell, M. J. (2024). In Vivo mRNA CAR T Cell Engineering via Targeted Ionizable Lipid Nanoparticles with Extrahepatic Tropism. *Small*, *20*(11), e2304378. https://doi.org/10.1002/smll.202304378

Chen, X., Yang, M., Chen, Y., Zhang, Y., Wang, S., Meng, J., Zhu, Z., Li, W., Wei, W., Ping, Y., & Liang, T. (2026). Genetic engineering of systemically injectable oncolytic viruses for pyroptosis-accelerated cancer virotherapy. *Nature Cancer*, *7*(1), 207–223. https://doi.org/10.1038/s43018-025-01078-y

Cheng, Z., Li, M., Dey, R., & Chen, Y. (2021). Nanomaterials for cancer therapy: current progress and perspectives. *J Hematol Oncol*, *14*(1), 85. https://doi.org/10.1186/s13045-021-01096-0

de Vlieger, J. S. B., Crommelin, D. J. A., Tyner, K., Drummond, D. C., Jiang, W., McNeil, S. E., Neervannan, S., Crist, R. M., & Shah, V. P. (2019). Report of the AAPS Guidance Forum on the FDA Draft Guidance for Industry: "Drug Products, Including Biological Products, that Contain Nanomaterials". *Aaps j*, *21*(4), 56. https://doi.org/10.1208/s12248-019-0329-7

Elsharkasy, O. M., Nordin, J. Z., Hagey, D. W., de Jong, O. G., Schiffelers, R. M., Andaloussi, S. E., & Vader, P. (2020). Extracellular vesicles as drug delivery systems: Why and how? *Adv Drug Deliv Rev*, *159*, 332–343. https://doi.org/10.1016/j.addr.2020.04.004

Estapé Senti, M., de Jongh, C. A., Dijkxhoorn, K., Verhoef, J. J. F., Szebeni, J., Storm, G., Hack, C. E., Schiffelers, R. M., Fens, M. H., & Boross, P. (2022). Anti-PEG antibodies compromise the integrity of PEGylated lipid-based nanoparticles via complement. *J Control Release*, *341*, 475–486. https://doi.org/10.1016/j.jconrel.2021.11.042

Faria, M., Björnmalm, M., Thurecht, K. J., Kent, S. J., Parton, R. G., Kavallaris, M., Johnston, A. P. R., Gooding, J. J., Corrie, S. R., Boyd, B. J., Thordarson, P., Whittaker, A. K., Stevens, M. M., Prestidge, C. A., Porter, C. J. H., Parak, W. J., Davis, T. P., Crampin, E. J., & Caruso, F. (2018). Minimum information reporting in bio-nano experimental literature. *Nat Nanotechnol*, *13*(9), 777–785. https://doi.org/10.1038/s41565-018-0246-4

Gong, N., Sheppard, N. C., Billingsley, M. M., June, C. H., & Mitchell, M. J. (2021). Nanomaterials for T-cell cancer immunotherapy. *Nat Nanotechnol*, *16*(1), 25–36. https://doi.org/10.1038/s41565-020-00822-y

Gu, K., Liang, T., Hu, L., Zhao, Y., Ying, W., Zhang, M., Chen, Y., Liang, B., Lin, X., Zhang, Y., Wu, H., Wang, M., Zhu, Y., Wang, W., Zhang, Y., Zuo, C., Du, Z., Zhang, P., Song, J.,…Tan, W. (2025). Intraperitoneal programming of tailored CAR macrophages via

mRNA lipid nanoparticle to boost cancer immunotherapy. *Nat Commun*, *17*(1), 941. https://doi.org/10.1038/s41467-025-67674-9

Gustafson, H. H., Holt-Casper, D., Grainger, D. W., & Ghandehari, H. (2015). Nanoparticle Uptake: The Phagocyte Problem. *Nano Today*, *10*(4), 487–510. https://doi.org/10.1016/j.nantod.2015.06.006

Hingorani, S. R., Wang, L., Multani, A. S., Combs, C., Deramaudt, T. B., Hruban, R. H., Rustgi, A. K., Chang, S., & Tuveson, D. A. (2005). Trp53R172H and KrasG12D cooperate to promote chromosomal instability and widely metastatic pancreatic ductal adenocarcinoma in mice. *Cancer Cell*, *7*(5), 469–483. https://doi.org/10.1016/j.ccr.2005.04.023

Hou, X., Zaks, T., Langer, R., & Dong, Y. (2021). Lipid nanoparticles for mRNA delivery. *Nat Rev Mater*, *6*(12), 1078–1094. https://doi.org/10.1038/s41578-021-00358-0

Kulkarni, J. A., Witzigmann, D., Thomson, S. B., Chen, S., Leavitt, B. R., Cullis, P. R., & van der Meel, R. (2021). The current landscape of nucleic acid therapeutics. *Nat Nanotechnol*, *16*(6), 630–643. https://doi.org/10.1038/s41565-021-00898-0

Li, M., Yang, Y., Yang, Z., Kang, L., Ma, Z., Luo, J., Fan, Z., Tian, X., Deng, Y., Ke, H., Liu, F., Tang, Y., Hu, J., Chen, H., & Yang, T. (2025). J-Aggregated indocyanine green-loaded exosomes enable photoactivatable cytoplasmic delivery of STING agonist for targeted pancreatic cancer immunotherapy. *Nano Today*, *62*, 102727. https://doi.org/https://doi.org/10.1016/j.nantod.2025.102727

Li, Y., Liu, B., Li, J., Ruan, C., Li, Z., Li, Y., Yang, Y., Li, S., Liu, X., Li, L., Li, L., Fan, K., Dang, X., & Cui, Y. (2025). An 'AND' Logic Gate Nanoreactor for Metabolic Remodeling in Starvation-Ferroptosis-Immunotherapy of Pancreatic Cancer. *Advanced Functional Materials*, *35*(51), e02221. https://doi.org/https://doi.org/10.1002/adfm.202502221

Liu, J., Li, B., Li, L., Ming, X., & Xu, Z. P. (2024). Advances in Nanomaterials for Immunotherapeutic Improvement of Cancer Chemotherapy. *Small*, *20*(38), e2403024. https://doi.org/10.1002/smll.202403024

Ma, R., Li, Z., Chiocca, E. A., Caligiuri, M. A., & Yu, J. (2023). The emerging field of oncolytic virus-based cancer immunotherapy. *Trends Cancer*, *9*(2), 122–139. https://doi.org/10.1016/j.trecan.2022.10.003

May, J. N., Moss, J. I., Mueller, F., Golombek, S. K., Biancacci, I., Rizzo, L., Elshafei, A. S., Gremse, F., Pola, R., Pechar, M., Etrych, T., Becker, S., Trautwein, C., Bülow, R. D., Boor, P., Knuechel, R., von Stillfried, S., Storm, G., Puri, S.,…Lammers, T. (2024). Histopathological biomarkers for predicting the tumour accumulation of nanomedicines. *Nat Biomed Eng*, *8*(11), 1366–1378. https://doi.org/10.1038/s41551-024-01197-4

Mehta, M., Bui, T. A., Yang, X., Aksoy, Y., Goldys, E. M., & Deng, W. (2023). Lipid-Based Nanoparticles for Drug/Gene Delivery: An Overview of the Production Techniques and Difficulties Encountered in Their Industrial Development. *ACS Mater Au*, *3*(6), 600–619. https://doi.org/10.1021/acsmaterialsau.3c00032

Melamed, J. R., Yerneni, S. S., Arral, M. L., LoPresti, S. T., Chaudhary, N., Sehrawat, A., Muramatsu, H., Alameh, M. G., Pardi, N., Weissman, D., Gittes, G. K., & Whitehead, K. A. (2023). Ionizable lipid nanoparticles deliver mRNA to pancreatic β cells via

macrophage-mediated gene transfer. *Sci Adv*, *9*(4), eade1444. https://doi.org/10.1126/sciadv.ade1444

Meng, Q., Ding, B., Ma, P., & Lin, J. (2025). Inorganic Nanobiomaterials Boost Tumor Immunotherapy: Strategies and Applications. *Acc Chem Res*, *58*(8), 1210–1223. https://doi.org/10.1021/acs.accounts.4c00843

Miao, Y., Li, L., Wang, Y., Wang, J., Zhou, Y., Guo, L., Zhao, Y., Nie, D., Zhang, Y., Zhang, X., & Gan, Y. (2024). Regulating protein corona on nanovesicles by glycosylated polyhydroxy polymer modification for efficient drug delivery. *Nat Commun*, *15*(1), 1159. https://doi.org/10.1038/s41467-024-45254-7

Mitchell, M. J., Billingsley, M. M., Haley, R. M., Wechsler, M. E., Peppas, N. A., & Langer, R. (2021). Engineering precision nanoparticles for drug delivery. *Nat Rev Drug Discov*, *20*(2), 101–124. https://doi.org/10.1038/s41573-020-0090-8

Monopoli, M. P., Aberg, C., Salvati, A., & Dawson, K. A. (2012). Biomolecular coronas provide the biological identity of nanosized materials. *Nat Nanotechnol*, *7*(12), 779–786. https://doi.org/10.1038/nnano.2012.207

Mura, S., Nicolas, J., & Couvreur, P. (2013). Stimuli-responsive nanocarriers for drug delivery. *Nat Mater*, *12*(11), 991–1003. https://doi.org/10.1038/nmat3776

Noman, M. Z., Desantis, G., Janji, B., Hasmim, M., Karray, S., Dessen, P., Bronte, V., & Chouaib, S. (2014). PD-L1 is a novel direct target of HIF-1α, and its blockade under hypoxia enhanced MDSC-mediated T cell activation. *J Exp Med*, *211*(5), 781–790. https://doi.org/10.1084/jem.20131916

Özdemir, B. C., Pentcheva-Hoang, T., Carstens, J. L., Zheng, X., Wu, C. C., Simpson, T. R., Laklai, H., Sugimoto, H., Kahlert, C., Novitskiy, S. V., De Jesus-Acosta, A., Sharma, P., Heidari, P., Mahmood, U., Chin, L., Moses, H. L., Weaver, V. M., Maitra, A., Allison, J. P.,…Kalluri, R. (2014). Depletion of carcinoma-associated fibroblasts and fibrosis induces immunosuppression and accelerates pancreas cancer with reduced survival. *Cancer Cell*, *25*(6), 719–734. https://doi.org/10.1016/j.ccr.2014.04.005

Percie du Sert, N., Hurst, V., Ahluwalia, A., Alam, S., Avey, M. T., Baker, M., Browne, W. J., Clark, A., Cuthill, I. C., Dirnagl, U., Emerson, M., Garner, P., Holgate, S. T., Howells, D. W., Karp, N. A., Lazic, S. E., Lidster, K., MacCallum, C. J., Macleod, M.,…Würbel, H. (2020). The ARRIVE guidelines 2.0: Updated guidelines for reporting animal research. *PLoS Biol*, *18*(7), e3000410. https://doi.org/10.1371/journal.pbio.3000410

Rahib, L., Smith, B. D., Aizenberg, R., Rosenzweig, A. B., Fleshman, J. M., & Matrisian, L. M. (2014). Projecting cancer incidence and deaths to 2030: the unexpected burden of thyroid, liver, and pancreas cancers in the United States. *Cancer Res*, *74*(11), 2913–2921. https://doi.org/10.1158/0008-5472.Can-14-0155

Ramanathan, R. K., Korn, R. L., Raghunand, N., Sachdev, J. C., Newbold, R. G., Jameson, G., Fetterly, G. J., Prey, J., Klinz, S. G., Kim, J., Cain, J., Hendriks, B. S., Drummond, D. C., Bayever, E., & Fitzgerald, J. B. (2017). Correlation between Ferumoxytol Uptake in Tumor Lesions by MRI and Response to Nanoliposomal Irinotecan in Patients with Advanced Solid Tumors: A Pilot Study. *Clin Cancer Res*, *23*(14), 3638–3648. https://doi.org/10.1158/1078-0432.Ccr-16-1990

Rosenblum, D., Joshi, N., Tao, W., Karp, J. M., & Peer, D. (2018). Progress and challenges towards targeted delivery of cancer therapeutics. *Nat Commun*, *9*(1), 1410. https://doi.org/10.1038/s41467-018-03705-y

Rurik, J. G., Tombácz, I., Yadegari, A., Méndez Fernández, P. O., Shewale, S. V., Li, L., Kimura, T., Soliman, O. Y., Papp, T. E., Tam, Y. K., Mui, B. L., Albelda, S. M., Puré, E., June, C. H., Aghajanian, H., Weissman, D., Parhiz, H., & Epstein, J. A. (2022). CAR T cells produced in vivo to treat cardiac injury. *Science*, *375*(6576), 91–96. https://doi.org/10.1126/science.abm0594

Sabnis, S., Kumarasinghe, E. S., Salerno, T., Mihai, C., Ketova, T., Senn, J. J., Lynn, A., Bulychev, A., McFadyen, I., Chan, J., Almarsson, Ö., Stanton, M. G., & Benenato, K. E. (2018). A Novel Amino Lipid Series for mRNA Delivery: Improved Endosomal Escape and Sustained Pharmacology and Safety in Non-human Primates. *Mol Ther*, *26*(6), 1509–1519. https://doi.org/10.1016/j.ymthe.2018.03.010

Salvati, A., Pitek, A. S., Monopoli, M. P., Prapainop, K., Bombelli, F. B., Hristov, D. R., Kelly, P. M., Åberg, C., Mahon, E., & Dawson, K. A. (2013). Transferrin-functionalized nanoparticles lose their targeting capabilities when a biomolecule corona adsorbs on the surface. *Nat Nanotechnol*, *8*(2), 137–143. https://doi.org/10.1038/nnano.2012.237

Schoenmaker, L., Witzigmann, D., Kulkarni, J. A., Verbeke, R., Kersten, G., Jiskoot, W., & Crommelin, D. J. A. (2021). mRNA-lipid nanoparticle COVID-19 vaccines: Structure and stability. *Int J Pharm*, *601*, 120586. https://doi.org/10.1016/j.ijpharm.2021.120586

Selvanesan, B. C., Chandra, D., Quispe-Tintaya, W., Jahangir, A., Patel, A., Meena, K., Alves Da Silva, R. A., Friedman, M., Gabor, L., Khouri, O., Libutti, S. K., Yuan, Z., Li, J., Siddiqui, S., Beck, A., Tesfa, L., Koba, W., Chuy, J., McAuliffe, J. C.,…Gravekamp, C. (2022). Listeria delivers tetanus toxoid protein to pancreatic tumors and induces cancer cell death in mice. *Sci Transl Med*, *14*(637), eabc1600. https://doi.org/10.1126/scitranslmed.abc1600

Sethumadhavan, S., Silva, M., Philbrook, P., Nguyen, T., Hatfield, S. M., Ohta, A., & Sitkovsky, M. V. (2017). Hypoxia and hypoxia-inducible factor (HIF) downregulate antigen-presenting MHC class I molecules limiting tumor cell recognition by T cells. *PLoS One*, *12*(11), e0187314. https://doi.org/10.1371/journal.pone.0187314

Singh, P., Pandit, S., Balusamy, S. R., Madhusudanan, M., Singh, H., Amsath Haseef, H. M., & Mijakovic, I. (2025). Advanced Nanomaterials for Cancer Therapy: Gold, Silver, and Iron Oxide Nanoparticles in Oncological Applications. *Adv Healthc Mater*, *14*(4), e2403059. https://doi.org/10.1002/adhm.202403059

Szebeni, J., Simberg, D., González-Fernández, Á., Barenholz, Y., & Dobrovolskaia, M. A. (2018). Roadmap and strategy for overcoming infusion reactions to nanomedicines. *Nat Nanotechnol*, *13*(12), 1100–1108. https://doi.org/10.1038/s41565-018-0273-1

Tang, L., Zhang, A., Zhang, Z., Zhao, Q., Li, J., Mei, Y., Yin, Y., & Wang, W. (2022). Multifunctional inorganic nanomaterials for cancer photoimmunotherapy. *Cancer Commun (Lond)*, *42*(2), 141–163. https://doi.org/10.1002/cac2.12255

Van Cutsem, E., Tempero, M. A., Sigal, D., Oh, D. Y., Fazio, N., Macarulla, T., Hitre, E., Hammel, P., Hendifar, A. E., Bates, S. E., Li, C. P., Hingorani, S. R., de la Fouchardiere, C., Kasi, A., Heinemann, V., Maraveyas, A., Bahary, N., Layos, L., Sahai, V.,…Bullock, A. J. (2020). Randomized Phase III Trial of Pegvorhyaluronidase Alfa With

Nab-Paclitaxel Plus Gemcitabine for Patients With Hyaluronan-High Metastatic Pancreatic Adenocarcinoma. *J Clin Oncol*, *38*(27), 3185–3194. https://doi.org/10.1200/jco.20.00590

Voke, E., Arral, M. L., Squire, H. J., Lin, T. J., Zheng, L., Coreas, R., Lui, A., Iavarone, A. T., Pinals, R. L., Whitehead, K. A., & Landry, M. P. (2025). Protein corona formed on lipid nanoparticles compromises delivery efficiency of mRNA cargo. *Nat Commun*, *16*(1), 8699. https://doi.org/10.1038/s41467-025-63726-2

Wang, S., Wang, H., Drabek, A., Smith, W. S., Liang, F., & Huang, Z. R. (2024). Unleashing the Potential: Designing Antibody-Targeted Lipid Nanoparticles for Industrial Applications with CMC Considerations and Clinical Outlook. *Mol Pharm*, *21*(1), 4–17. https://doi.org/10.1021/acs.molpharmaceut.3c00735

Wedge, M. E., Jennings, V. A., Crupi, M. J. F., Poutou, J., Jamieson, T., Pelin, A., Pugliese, G., de Souza, C. T., Petryk, J., Laight, B. J., Boileau, M., Taha, Z., Alluqmani, N., McKay, H. E., Pikor, L., Khan, S. T., Azad, T., Rezaei, R., Austin, B.,…Ilkow, C. S. (2022). Virally programmed extracellular vesicles sensitize cancer cells to oncolytic virus and small molecule therapy. *Nat Commun*, *13*(1), 1898. https://doi.org/10.1038/s41467-022-29526-8

Welsh, J. A., Goberdhan, D. C. I., O'Driscoll, L., Buzas, E. I., Blenkiron, C., Bussolati, B., Cai, H., Di Vizio, D., Driedonks, T. A. P., Erdbrügger, U., Falcon-Perez, J. M., Fu, Q. L., Hill, A. F., Lenassi, M., Lim, S. K., Mahoney, M. G., Mohanty, S., Möller, A., Nieuwland, R.,…Witwer, K. W. (2024). Minimal information for studies of extracellular vesicles (MISEV2023): From basic to advanced approaches. *J Extracell Vesicles*, *13*(2), e12404. https://doi.org/10.1002/jev2.12404

Yang, D., Liu, J., Qian, H., & Zhuang, Q. (2023). Cancer-associated fibroblasts: from basic science to anticancer therapy. *Exp Mol Med*, *55*(7), 1322–1332. https://doi.org/10.1038/s12276-023-01013-0

Yen, Y. T., Zhang, Z., Chen, A., Qiu, Y., Liu, Q., Wang, Q., Li, C., Wang, C., Qian, X., Shao, J., Meng, F., Yu, L., Liu, B., & Li, R. (2025). Enzymatically responsive nanocarriers targeting PD-1 and TGF-β pathways reverse immunotherapeutic resistance and elicit robust therapeutic efficacy. *J Nanobiotechnology*, *23*(1), 124. https://doi.org/10.1186/s12951-025-03129-z

Yu, Z., Shen, X., Yu, H., Tu, H., Chittasupho, C., & Zhao, Y. (2023). Smart Polymeric Nanoparticles in Cancer Immunotherapy. *Pharmaceutics*, *15*(3). https://doi.org/10.3390/pharmaceutics15030775

Zeng, Y., Tao, Y., Du, G., Huang, T., Chen, S., Fan, L., & Zhang, N. (2025). Advances in the mechanisms of HIF-1α-enhanced tumor glycolysis and its relation to dedifferentiation. *Prog Biophys Mol Biol*, *197*, 1–10. https://doi.org/10.1016/j.pbiomolbio.2025.05.003

Zhu, X., & Li, S. (2023). Nanomaterials in tumor immunotherapy: new strategies and challenges. *Mol Cancer*, *22*(1), 94. https://doi.org/10.1186/s12943-023-01797-9
